# Screening of strawberry (*Fragaria × ananassa* Duch.) cultivars for drought tolerance based on physiological and biochemical responses under PEG-induced stress

**DOI:** 10.3389/fpls.2025.1655320

**Published:** 2025-09-15

**Authors:** Şule Hilal Attar, Duygu Ayvaz Sonmez, Azam Akbari, Doğan Ergün, Ömer Faruk Bilgin, Betül Yeşil, Merve Onur Bozkurt, Hayriye Yıldız Daşgan, Boran İkiz, Salih Kafkas, Bruno Mezzetti, Nesibe Ebru Kafkas

**Affiliations:** ^1^ Department of Horticulture, University of Çukurova, Faculty of Agriculture, Adana, Türkiye; ^2^ Yaltir Agricultural Products Inc., Adana, Türkiye; ^3^ Department of Horticulture, University of Siirt, Faculty of Agriculture, Siirt, Türkiye; ^4^ Department of Silviculture, Faculty of Forestry, Istanbul University-Cerrahpaşa, Bahçeköy, Sariyer, Istanbul, Türkiye; ^5^ Department of Agricultural, Food and Environmental Sciences, Universita Politecnica delle Marche, Ancona, Italy

**Keywords:** strawberry, drought stress, PEG-induced stress, physiological traits, biochemical markers, cultivar-specific response, antioxidant activity

## Abstract

Climate change-driven water scarcity poses increasing challenges to strawberry production worldwide. This study evaluated drought tolerance mechanisms in fifteen strawberry (*Fragaria* × *ananassa Duch*.) cultivars in the first year and seven selected cultivars in the second year, including Turkish local varieties, using polyethylene glycol (PEG)-induced drought stress in controlled greenhouse conditions. Key physiological and biochemical parameters were assessed, including relative water content (RWC), chlorophyll content, Photosynthetic Quantum Yield, leaf dry matter content (LDMC), sugar composition, and antioxidant capacity. Drought stress significantly reduced RWC by 13.1% in year one and 7.5% in year two, while chlorophyll content declined by 4.8% and 6.6%, respectively. Photosynthetic Quantum Yield decreased by 7.9% and 12.2% across the two years. Conversely, LDMC increased by approximately 16% in both years, indicating morphological adaptation to water deficit. The most striking response was carbohydrate accumulation, particularly in year one, where total sugar content in 'Sweet Charlie' increased from 3.48% to 28.42%, and 'Monterey' showed an increase from 2.69% to 22.90%. Year two exhibited more moderate sugar responses but stronger correlations between sugar content and RWC (glucose: r = 0.93), suggesting refined osmotic adjustment mechanisms. Antioxidant activity increased across all genotypes under stress, with 'Festival' and 'Sabrina' achieving over 90% antioxidant activity. Principal component analysis and hierarchical clustering effectively discriminated drought-tolerant from sensitive cultivars. 'Brilliance' demonstrated superior drought tolerance, maintaining high RWC (89.2%) and stable sugar metabolism under stress conditions. In contrast, 'Calderon' and 'Plared' showed significant declines in physiological performance. The study reveals cultivar-specific drought response strategies, with sugar accumulation, chlorophyll stability, and antioxidant capacity serving as reliable screening markers. The identified physiological benchmarks and tolerant genotypes ('Brilliance', 'Festival', 'Sweet Charlie', and local variety 'Arnavutkِy') provide valuable resources for breeding programs targeting enhanced drought resilience. These findings contribute to understanding strawberry adaptation mechanisms under water stress and offer practical tools for sustainable production in water-limited environments.

## 1 Introduction

Climate change poses an escalating threat to global agricultural systems, with water scarcity emerging as one of the most critical constraints on crop productivity and food security. Projections from contemporary climate models suggest increasingly severe, frequent, and prolonged drought events across diverse agroecological regions (Dai, 2013). The intricate interplay between rising global temperatures and altered precipitation patterns continues to disrupt traditional cropping systems, challenging conventional water management practices (Trenberth, 2011).

Strawberry (*Fragaria × ananassa* Duch.), a globally cultivated high-value horticultural berry crop, is particularly susceptible to drought due to its shallow root architecture, high leaf area index, and continuous water demands during critical developmental stages (Ghaderi and Siosemardeh, 2011). Under water deficit conditions, strawberry plants undergo complex morphological and biochemical changes including reduced photosynthetic efficiency, overproduction of reactive oxygen species (ROS), and shifts in osmotic regulation mechanisms (Klamkowski and Treder, 2008). Despite its sensitivity to drought, global strawberry production has remained robust, with total output reaching approximately 9.56 million metric tons in 2022 (FAO, 2023). The economic significance of the crop is equally remarkable, with the global strawberry market valued at nearly USD 20.22 billion in 2023 (Zion Market Research, 2023). In addition to its economic value, strawberries are rich in bioactive compounds, including vitamin C, anthocyanins, and antioxidants, contributing to their increasing popularity in the functional food sector (Giampieri et al., 2015).

Like other fruit species drought stress profoundly influences both yield and fruit quality in strawberry. Even moderate water deficit can lead to rapid declines in photosynthetic rate and significant yield losses (Martínez-Ferri et al., 2016; Cordoba-Novoa et al., 2021). Terry et al. (2008) demonstrated that deficit irrigation could reduce berry weight by up to 1.4-fold compared to fully irrigated controls. The plant’s response to drought is inherently dynamic, involving a coordinated activation of physiological and molecular defense pathways. Fang and Xiong (2015) outlined four fundamental drought resistance strategies: avoidance, tolerance, escape, and recovery each reflecting distinct adaptations that support survival in water-limited environments.

Water scarcity triggers a cascade of physiological processes in plants. One of the earliest responses involves abscisic acid (ABA) mediated stomatal closure to minimize transpirational water loss (Yang Y.J, et al., 2021; Tombesi et al., 2015). While this response conserves water, it also limits CO_2_ diffusion, reducing photosynthetic efficiency and leading to photoinhibition and chlorophyll degradation (Chaves et al., 2009; Muhammad et al., 2021). Furthermore, drought conditions often lead to the overproduction of ROS, which disrupt cellular homeostasis and damage membranes, proteins, and DNA (Li et al., 2022; Panda et al., 2024). Plants counteract these effects by accumulating compatible solutes such as sugars, amino acids, and ions which help maintain osmotic balance and water uptake under stress (Ashraf and Harris, 2004; Chen and Jiang, 2010).

At the molecular level, drought signaling involves key molecules including ABA, Ca^2+^, nitric oxide (NO), and secondary messengers, which modulate gene expression and physiological responses (Yang X, et al., 2021). Morphologically, plants adapt through reductions in leaf area, increased leaf thickness, and higher leaf tissue density traits that enhance water conservation and stress tolerance.

Importantly, the wide genetic diversity among cultivated strawberry genotypes offers valuable opportunities to explore differential drought responses. The cultivated octoploid strawberry (2n = 8x = 56) possesses considerable genetic plasticity due to its interspecific hybrid origin, enabling a broad range of physiological adaptations (Folta and Barbey, 2019). Zahedi et al. (2020) highlighted that drought stress adversely affects strawberry morphology, enzyme activities, and developmental processes, underscoring the need for cultivar-specific evaluations.

In the present study, sixteen strawberry cultivars including fourteen commercially cultivated varieties and two locally adapted Turkish cultivars were systematically screened under drought stress induced by Polyethylene Glycol (PEG) in greenhouse conditions. Through a comprehensive physiological and biochemical approach, key traits such as chlorophyll content, photosynthetic quantum yield, antioxidant enzyme activity, carbohydrate accumulation, and plant water status were evaluated. The primary objectives of this research were to: (1) assess genotypic variability in drought tolerance; (2) identify reliable physiological and biochemical markers associated with drought adaptation; and (3) generate cultivar-specific response profiles to support future breeding programs targeting enhanced drought resilience in strawberry. This integrative analysis aims to provide both practical insights and scientific foundations for improving strawberry performance under water-limited conditions.

## 2 Materials and methods

### 2.1 Experimental location

The study was conducted in the plastic greenhouse of the Research and Experimental Center of Çukurova University, located in Adana provinces in Turkey (Latitude: 37.0392°N, Longitude: 35.3710°E; Elevation: 127 meters above sea level). The region has a Mediterranean climate characterized by hot, dry summers and mild, wet winters. The greenhouse used in this study was a tunnel-type structure equipped with a partial environmental control system, allowing passive ventilation and regulation of temperature and humidity. The location provided consistent daylight exposure during the experimental periods, supporting uniform plant growth conditions. All experiments were carried out within the same facility in both years (2022 and 2024) to ensure consistency across environmental variables.

### 2.2 Plant materials and growing conditions

Fifteen strawberry (*Fragaria × ananassa* Duch.) cultivars were used in the first year of the study. These included fourteen commercially cultivated international varieties (San Andreas, Sabrina, Brilliance, Beauty, Festival, Ananas, Calderon, Camarosa, Fronteras, Merced, Monterey, Plared, Portola, and Sweet Charlie) and one local Turkish cultivar, Arnavutköy. Seven cultivars were selected for further evaluation in the second year. This set included five commercial cultivars (San Andreas, Sabrina, Brilliance, Beauty, and Festival) and two local Turkish genotypes (Arnavutköy and Ereğli). The selections used in the second year were chosen based on contrasting physiological and biochemical responses observed in the first year. All plants were re-established in fresh cocopeat substrate at the beginning of each growing season to ensure consistency in root development and nutrient availability.

Each plant was grown individually in 5-liter plastic pots filled with a soil-based growing medium consisting of peat moss, perlite, and sand in a 2:1:1 ratio (v/v/v), adjusted to a pH of 5.5 – 6.5. To prevent inter-plant competition and ensure uniform light and air exposure, pots were spaced 30 cm apart throughout the experiment. Greenhouse temperature was maintained at 24 ± 2 °C during the day and 18 ± 2 °C at night, with relative humidity between 60% and 70%.

### 2.3 Experimental design and treatments

The experiment was conducted over two consecutive years (2022 and 2024) following a randomized complete block design (RCBD) with three replications per treatment. In the first year (2022), plants were subjected to two irrigation treatments: 100% irrigation (control) and drought stress treatment induced by polyethylene glycol (PEG - 6000). For the control treatment, soil moisture was maintained at field capacity (35 – 40% volumetric water content), while drought stress was simulated by irrigating plants with a 20% (w/v) PEG - 6000 solution, corresponding to an osmotic potential of approximately -0.5 MPa.

Each experimental unit consisted of a single plant per replication, resulting in a total of 96 plants in 2022 (16 entries × 2 treatments × 3 replications) and 42 plants in 2024 (7 entries × 2 treatments × 3 replications). The stress period lasted for eight weeks during the plants’ active growth stage. Standard cultural practices, including fertilization, pest control, and pruning, were applied uniformly to all plants throughout the experiment.

### 2.4 Data collection and measurements

#### 2.4.1 Morphological parameters

##### 2.4.1.1 Leaf number

Leaf number was determined by counting the total number of fully expanded leaves per plant at the time of measurement.

##### 2.4.1.2 Leaf dry matter content

Fresh leaves were weighed after collection for fresh weight (FW). The samples were dried in an oven at 70 °C for 72 hours, or until they reached a constant weight, and then reweighed to determine dry weight (DW). Leaf dry matter content was calculated as (DW/FW) 100%.

#### 2.4.2 Physiological parameters

##### 2.4.2.1 Leaf temperature

Leaf temperature was measured using a Fluke 62 Max infrared thermometer between 08:00 and 10:00 a.m. to minimize environmental variation. The device was held 0.5 m above the canopy, and each leaf was measured three times to obtain an average. This method enables non-contact assessment of plant physiological responses under drought stress (González-Villagra et al., 2024; Barai et al., 2022).

##### 2.4.2.2 Chlorophyll content

The non-destructive estimation of leaf chlorophyll content utilized a chlorophyll meter (SPAD - 502 Plus, Konica Minolta, Japan). Five measurements were made per leaf (to prevent measuring over major veins) on five leaves per plant and averaged. SPAD values were converted into mmol m^-2^ of chlorophyll content using the following calibration equation, developed by Markwell et al. (1995), which converts SPAD values to chlorophyll content: Chlorophyll content = 10^ (SPAD × 0.0265 + 0.9).

##### 2.4.2.3 Photosynthetic quantum yield

Chlorophyll fluorescence was measured using a portable fluorometer (MINI-PAM-II, Walz, Germany) following Baker (2008). Measurements were performed on five fully expanded leaves per plant (three plants per treatment) from the middle section of the shoots, between 08:00–10:00 h to avoid potential effects of midday photoinhibition. The effective quantum yield of PSII (ΦPSII) was calculated as (Fm′ – Fs)/Fm′, where Fs is steady-state fluorescence and Fm′ is maximum fluorescence under actinic light. Lower ΦPSII values were interpreted as an indication of greater stress impact on photosynthetic efficiency.

##### 2.4.2.4 Leaf relative water content

Relative Water Content (RWC) was determined following the method of Turner (1997), with modifications by Wilkinson et al. (2001). Freshly detached leaves (~1 g) were weighed for fresh weight (FW), then submerged in water overnight in the dark to obtain turgid weight (TW). Subsequently, leaves were dried at 80 °C for 24 hours to determine dry weight (DW). RWC was calculated using the formula:


RWC (%)=[(FW-DW)/(TW-DW)]×100

This method provides a reliable estimate of plant water status under drought conditions.

#### 2.4.3 Biochemical parameters

##### 2.4.3.1 Preparation of the extracts

The leaves were pulverized and powdered finely using an analytical mill. 100 mg of the leaf powder was mixed with 10 mL ultra-pure water. The solution was subjected to a 1-hour ultrasound treatment followed by centrifugation at 10,000 rpm. The clear supernatant liquid was poured gently into vials for analysis. 1 mL of the supernatant was diluted 50 times for analysis and kept for other tests.

##### 2.4.3.2 Determination of antioxidant activity and free radical scavenging

###### 2.4.3.2.1 DPPH radical scavenging activity

The antioxidant and free radical scavenging activity of the dried leaf extracts was assessed using the DPPH assay, following the method described by Pękal and Pyrzynska (2013). In this procedure, 0.1 mL of extract was mixed with 2.4 mL of DPPH solution (9 × 10^-5^ mol/L) prepared in methanol. After thorough mixing, the reaction mixture was incubated in the dark at room temperature for 30 minutes. Absorbance was then recorded at 518 nm using a spectrophotometer, with methanol as the blank. The percentage of DPPH radical scavenging activity (DPPH_RSA) was calculated using the corresponding formula.


DPPH_RSA (%)=[(A_blank-A_extract)/A_blank]×100

where A_control is the absorbance of DPPH solution in the absence of extract, and A_sample is the absorbance of the reaction mixture with extract.

###### 2.4.3.2.2 DPPH inhibition

The DPPH radical scavenging activity (DPPH_inh) was also evaluated using the same assay, with absorbance measured at 518 nm. The percentage of inhibition, reflecting the sample’s capacity to neutralize DPPH free radicals, was calculated using the corresponding formula:


DPPH_inh (%)=[(A_control-A_sample)/A_control]×100

Both results were expressed as percentages (%), representing the antioxidant potential of the dried leaf extracts.

##### 2.4.3.3 Individual sugar determination

The concentrations of sucrose, glucose, fructose, and total sugars were determined using a Shimadzu LC 20A VP HPLC system (Kyoto, Japan) equipped with a refractive index detector. Sugar separation was carried out on a reverse-phase Ultrasphere Coregel-87 C column (300 mm × 7.8 mm, 5 µm) maintained at 70 °C, with ultrapure water as the mobile phase at a constant flow rate of 0.6 mL/min under isocratic conditions. A 20 µL sample volume was injected for each run. Sugar contents were quantified based on calibration with external standards and expressed as a percentage of dry weight (DW).

### 2.5 Statistical analysis

Statistical analyses were performed using analysis of variance (ANOVA) in SAS software (version 9.4; SAS Institute Inc., USA), considering the effects of cultivar, treatment, year, and their interactions. Significant differences among treatments were determined using Duncan’s multiple range test at p< 0.05. The results are indicated by different lowercase letters in the tables and figures. Pearson correlation coefficients were calculated to assess relationships among the measured traits. Multivariate analyses, including principal component analysis (PCA) and hierarchical clustering with heatmap visualization, were conducted using R software (version 4.4.3; R Foundation for Statistical Computing) to identify patterns and illustrate associations between treatments and parameters.

## 3 Results and discussion

### 3.1 Analysis of variance

To evaluate variability in drought responses, separate ANOVAs were conducted for each year (**Supplementary Tables S2**, **S3**), and a combined ANOVA was performed for the six cultivars common to both years (**Supplementary Table S1**). This approach enabled assessment of seasonal variation and genotype stability under stress conditions. The combined analysis revealed highly significant effects (P< 0.001) of year, cultivar, and treatment on most physiological and biochemical traits. Year effects were especially evident in leaf temperature, chlorophyll content, and relative water content, indicating a strong environmental influence. Cultivar effects were consistently significant across all traits, with the greatest variation observed in leaf number and total sugar content, reflecting notable genetic diversity. Treatment effects (drought vs. well-watered) were also highly significant for most parameters, particularly sugar metabolism traits (sucrose, glucose, and fructose), though leaf number showed non-significant differences in some interactions. Significant cultivar × treatment interactions highlighted genotype-specific drought responses, especially in sugar content and antioxidant-related traits, suggesting different biochemical adaptation strategies. Year × treatment interactions were also significant for several parameters, particularly those related to sugar metabolism, confirming that drought responses varied between seasons. The consistency of cultivar and treatment effects across years, along with significant three-way interactions (cultivar × treatment × year) for biochemical traits, underscores the complexity of drought adaptation and supports the use of multi-year evaluations in breeding drought-resilient strawberry cultivars.

### 3.2 Physiological responses

#### 3.2.1 Leaf temperature response

Variations in leaf temperature between the two years were substantial, primarily due to seasonal differences in measurement timing. The experiment was conducted in the first year during winter (January), and in the second year during early summer (June), resulting in different baseline temperature conditions. Consequently, absolute leaf temperatures were naturally higher in the second year. Specifically, in the first year, average leaf temperature increased from 12.3 °C (control) to 14.7 °C under drought stress—a 19.5% rise—as shown in **Table 1**. In the second year, it increased from 23.7 °C to 26.2 °C, corresponding to a 10.5% increase (**Table 2**). This increase in leaf temperature under water deficit aligns with earlier studies showing that drought-induced stomatal closure reduces transpirational cooling, leading to thermal buildup in leaves (Peñuelas et al., 1992). Thus, leaf temperature serves as a sensitive, non-invasive indicator of physiological stress in strawberry. Interestingly, in the first year, cultivars Camarosa and Plared exhibited reduced leaf temperatures under drought stress—an exception to the general trend. This atypical response may be linked to cultivar-specific mechanisms such as increased leaf reflectance, altered leaf orientation, or distinct stomatal behavior. Similar traits have been described in wild strawberries; notably, Cao et al. (2022) identified structural, transcriptional, and epigenetic responses associated with drought tolerance in *Fragaria nilgerrensis*.

**Table 1:** Physiological traits of strawberry cultivars under PEG-induced drought stress and well-watered irrigation conditions in the first year.

Cultivar	Leaf temp (°C)		Chlorophyll (µmol/m)²		RWC (%)		Photosynthetic quantum yield		Leaf dry matter content (%)		Leaf number	
	100%	PEG	100%	PEG	100%	PEG	100%	PEG	100%	PEG	100%	PEG
Ananas	11.9 ± 0.20 ijk	15.73 ± 0.25 c	52.77 ± 2.16 bc	52.47 ± 1.19 bcde	80.77 ± 2.48 bcd	73.87 ± 2.47 fghi	0.62 ± 0.03 fg	0.61 ± 0.01 fghi	32.83 ± 0.12 g	37.13 ± 0.64 ab	19 ± 1.73 de	13.33 ± 1.53 ghij
Arnavutköy	11.77 ± 0.15 ijk	12.9 ± 0.10 fgh	45.53 ± 0.81 kl	43.1 ± 0.27 m	84.33 ± 4.83 ab	69.8 ± 0.56 ij	0.66 ± 0.02 bcde	0.63 ± 0.01 ef	30.07 ± 0.21 jk	34.7 ± 2.43 de	32 ± 2.00 a	24.67 ± 4.16 b
Beauty	11.17 ± 0.15 kl	14.07 ± 0.21 d	52.63 ± 1.16 bcd	47.3 ± 1.47 ijk	75.37 ± 0.38 efgh	61.1 ± 1.56 m	0.62 ± 0.04 fg	0.55 ± 0.04 jkl	35.7 ± 0.17 cd	35.7 ± 0.00 cd	15 ± 1.00 fgh	8 ± 1.73 mnop
Brilliance	10.53 ± 0.31 lm	11.77 ± 0.15 ijk	55 ± 0.70 a	55.33 ± 0.71 a	87.25 ± 0.85 a	67.9 ± 0.10 jkl	0.58 ± 0.03 ghij	0.58 ± 0.02 hijk	28.63 ± 1.35 lmn	32.37 ± 0.35 gh	9.67 ± 1.53 klmno	7 ± 1.00 op
Calderon	12.5 ± 0.10 ghi	13 ± 0.20 fg	46.93 ± 1.33 jk	43.73 ± 2.71 lm	70.1 ± 1.30 ij	68.03 ± 2.83 jk	0.62 ± 0.03 fg	0.55 ± 0.03 jkl	29.47 ± 0.23 jkl	33.4 ± 0.00 fg	14.67 ± 0.58 fghi	6 ± 1.73 p
Camarosa	14.33 ± 0.31 d	10.37 ± 0.91 m	50.73 ± 0.68 cdefgh	50.3 ± 0.66 efgh	75.5 ± 2.54 efgh	63.77 ± 1.68 lm	0.54 ± 0.02 kl	0.47 ± 0.02 m	29.17 ± 0.06 klm	37.5 ± 0.00 ab	20 ± 2.00 cde	12 ± 1.00 hijk
Festival	12.97 ± 0.59 fg	15.23 ± 0.21 c	51 ± 0.36 cdefgh	50.03 ± 0.75 fgh	79.37 ± 0.64 cde	64.73 ± 1.27 klm	0.74 ± 0.01 a	0.64 ± 0.03 cdef	29.2 ± 0.17 klm	33.3 ± 0.00 fg	21.67 ± 5.03 bcd	11.67 ± 1.53 hijkl
Fronteras	11.67 ± 0.55 jk	16.63 ± 0.35 b	53.9 ± 2.91 ab	49.8 ± 0.60 fgh	75.13 ± 1.64 efgh	73.67 ± 1.65 fghi	0.63 ± 0.03 ef	0.57 ± 0.00 jk	27.53 ± 0.31 nop	30.6 ± 1.11 ij	12 ± 1.73 hijk	7.67 ± 1.53 nop
Merced	12.4 ± 0.46 ghij	15.7 ± 0.52 c	49.37 ± 0.35 fghi	48.7 ± 0.66 hij	75.1 ± 1.13 efgh	69 ± 5.76 j	0.65 ± 0.02 bcdef	0.64 ± 0.01 cdef	31.5 ± 0.17 hi	35.57 ± 0.12 cd	9 ± 1.00 klmnop	7.33 ± 1.16 op
Monterey	12.17 ± 0.15 hij	17.1 ± 0.36 ab	51.17 ± 0.70 cdefg	47.03 ± 0.50 jk	77.47 ± 1.36 cdef	72.1 ± 2.50 ghij	0.67 ± 0.01 bcd	0.66 ± 0.01 bcde	25.07 ± 0.12 q	29.4 ± 0.35 jkl	13.33 ± 1.53 ghij	8.33 ± 0.58 lmnop
Plared	15.43 ± 0.38 c	11.9 ± 0.36 ijk	48.87 ± 0.72 ghij	50.43 ± 0.21 defgh	81.2 ± 3.12 bc	76.8 ± 2.10 def	0.54 ± 0.02 kl	0.61 ± 0.02 fgh	28.07 ± 0.12 mno	37.93 ± 1.34 a	23 ± 2.00 bc	9.33 ± 0.58 klmnop
Portolo	13.27 ± 0.15 ef	16.9 ± 0.10 ab	55.77 ± 1.63 a	50.43 ± 0.64 defgh	77.6 ± 1.18 cdef	63.87 ± 0.95 klm	0.68 ± 0.01 bc	0.66 ± 0.01 bcde	28.2 ± 0.27 lmno	34.23 ± 0.81 ef	15.67 ± 2.52 fg	11.33 ± 0.58 ijklm
Sabrina	11.93 ± 1.04 ijk	15.87 ± 0.35 c	50.7 ± 1.25 cdefgh	44.5 ± 0.76 lm	76.4 ± 3.41 defgh	57.2 ± 4.52 n	0.57 ± 0.01 ijk	0.51 ± 0.02 l	30.13 ± 0.67 jk	36.53 ± 0.91 bc	21.33 ± 2.52 cd	13.67 ± 0.58 ghij
San Andreas	12.27 ± 0.45 ghij	17.57 ± 0.51 a	55.47 ± 0.84 a	51.53 ± 1.62 cdef	76.43 ± 1.36 defgh	71.97 ± 1.16 hij	0.63 ± 0.01 def	0.56 ± 0.04 jk	26.53 ± 0.12 p	27 ± 0.30 op	14.67 ± 1.53 fghi	11 ± 1.73 jklmn
Sweet charlie	10.6 ± 0.60 lm	13.77 ± 0.25 de	54.53 ± 0.86 ab	52.37 ± 1.23 bcde	76.53 ± 0.85 defg	61.73 ± 2.29 m	0.68 ± 0.01 b	0.56 ± 0.01 jk	30.17 ± 0.31 jk	35.27 ± 0.45 de	17.33 ± 1.16 ef	10.33 ± 0.58 jklmno

Values followed by different lowercase letters within a column are significantly different at p< 0.05 (Duncan’s test).

**Table 2:** Physiological traits of strawberry cultivars under PEG-induced drought stress and well-watered irrigation conditions in the second year.

Cultivar	Leaf temp (°C)		Chlorophyll (µmol/m)²		RWC (%)		Photosynthetic quantum yield		Leaf dry matter content (%)		Leaf number	
	100%	PEG	100%	PEG	100%	PEG	100%	PEG	100%	PEG	100%	PEG
Arnavutköy	24.53 ± 0.21 ef	26 ± 0.80 cd	35.37 ± 1.33 de	35.27 ± 1.45 def	89.38 ± 3.81 abcd	84.14 ± 11.63 d	0.70 ± 0.01 e	0.62 ± 0.02 h	35.66 ± 1.07 ab	39.33 ± 0.43 a	15 ± 5.20 bc	6.67 ± 0.58 d
Beauty	23.07 ± 0.40 g	25.1 ± 0.36 de	40.57 ± 0.70 a	34.93 ± 0.55 ef	94.52 ± 4.24 ab	91.12 ± 1.21 abcd	0.67 ± 0.01 f	0.58 ± 0.01 i	25.81 ± 0.85 def	28.6 ± 0.58 cde	11.33 ± 1.16 c	4 ± 0.00 d
Brilliance	25.13 ± 0.25 de	26.37 ± 1.47 bc	37.03 ± 0.95 bcd	37.13 ± 2.16 bcd	94.17 ± 1.04 ab	89.19 ± 6.60 abcd	0.77 ± 0.01 ab	0.75 ± 0.01 bc	21.89 ± 1.37 f	25.07 ± 1.34 def	18 ± 3.00 ab	7 ± 0.00 d
Ereğli	25.13 ± 0.55 de	27.7 ± 0.27 a	37.53 ± 0.93 bc	35.8 ± 0.27 cde	96.07 ± 1.69 a	86.23 ± 8.28 bcd	0.77 ± 0.01 a	0.64 ± 0.00 g	26.01 ± 2.45 def	32.6 ± 5.91 bc	15 ± 0.00 bc	6.33 ± 0.58 d
Festival	21.27 ± 0.06 h	23.93 ± 0.15 efg	35.43 ± 1.33 de	33.27 ± 0.60 f	93.55 ± 1.10 abc	84.03 ± 3.35 d	0.75 ± 0.01 c	0.66 ± 0.01 f	22.95 ± 0.30 ef	29.86 ± 7.44 bcd	12 ± 3.00 c	4.33 ± 0.58 d
Sabrina	23.6 ± 0.36 fg	27.33 ± 1.27 ab	39.83 ± 0.78 a	33.8 ± 1.15 ef	91.67 ± 0.74 abcd	82.79 ± 0.81 d	0.77 ± 0.01 ab	0.71 ± 0.02 de	26.07 ± 6.05 def	31.36 ± 2.35 bcd	21.33 ± 3.06 a	7.33 ± 0.58 d
San Andreas	23.37 ± 0.21 fg	26.63 ± 1.11 abc	38.9 ± 0.46 ab	37.23 ± 1.37 bcd	91.73 ± 3.14 abcd	84.73 ± 3.39 cd	0.73 ± 0.01 d	0.63 ± 0.01 g	25.65 ± 3.39 def	27.85 ± 0.70 cdef	16.33 ± 2.08 b	5 ± 1.00 d

Values followed by different lowercase letters within a column are significantly different at p< 0.05 (Duncan’s test).

#### 3.2.2 Chlorophyll content

Drought stress led to a consistent reduction in chlorophyll content across most strawberry cultivars in both experimental years (P< 0.01). In the first year, the average chlorophyll content decreased from 51.6 μmol m^-2^ under control conditions to 49.1 μmol m^-2^ under PEG-induced stress, representing a 4.8% decline. Similarly, in the second year, it dropped from 37.8 μmol m^-2^ to 35.3 μmol m^-2^, corresponding to a 6.6% reduction. These trends are detailed in **Tables 1**, **2**, which summarize the cultivar-wise chlorophyll means under both irrigation regimes for each year.

**Figure 1:**
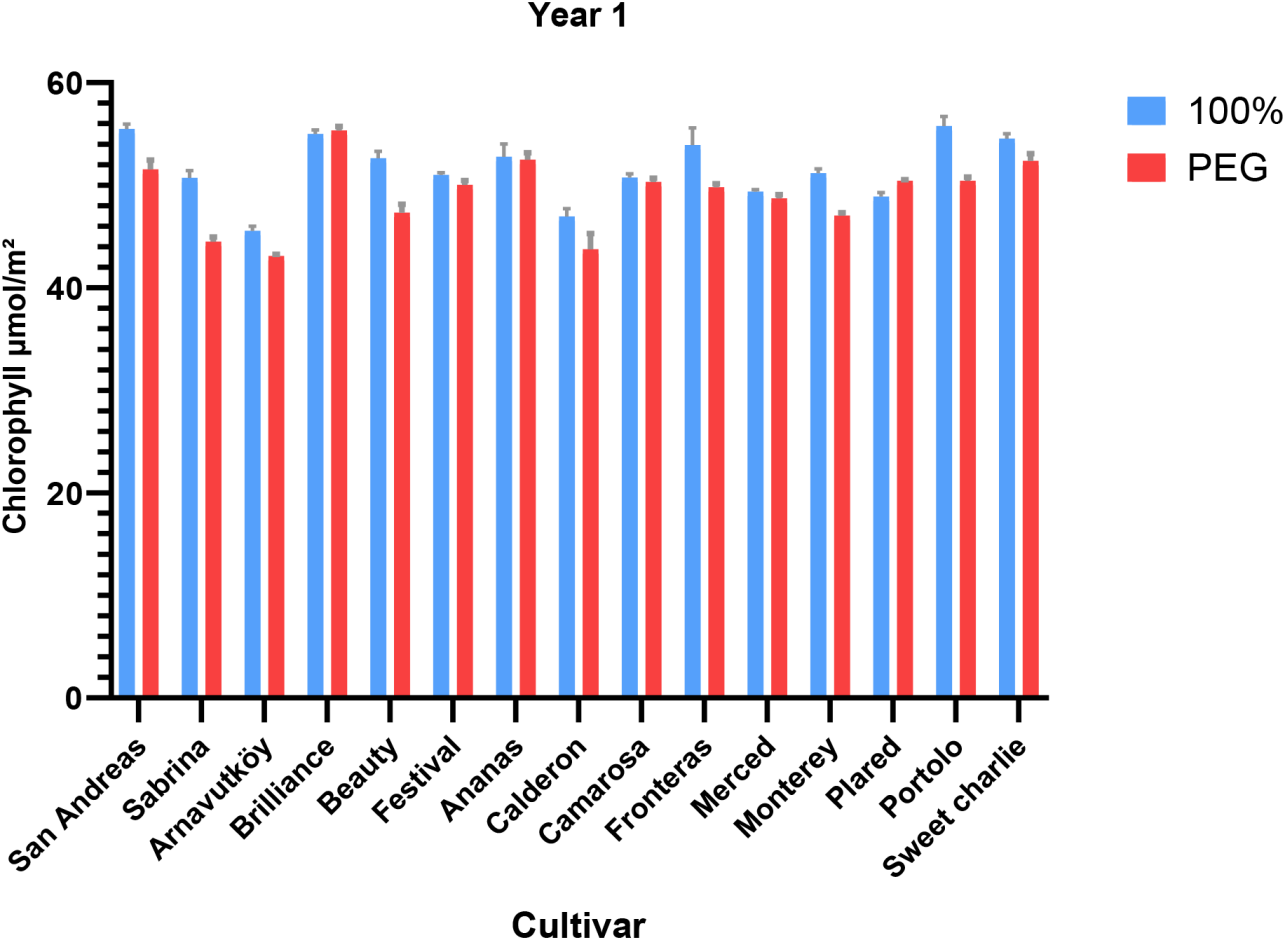
Chlorophyll content (μmol m^-2^) of strawberry cultivars under control and PEG-induced drought stress in Year 1 (2022).

**Figure 2:**
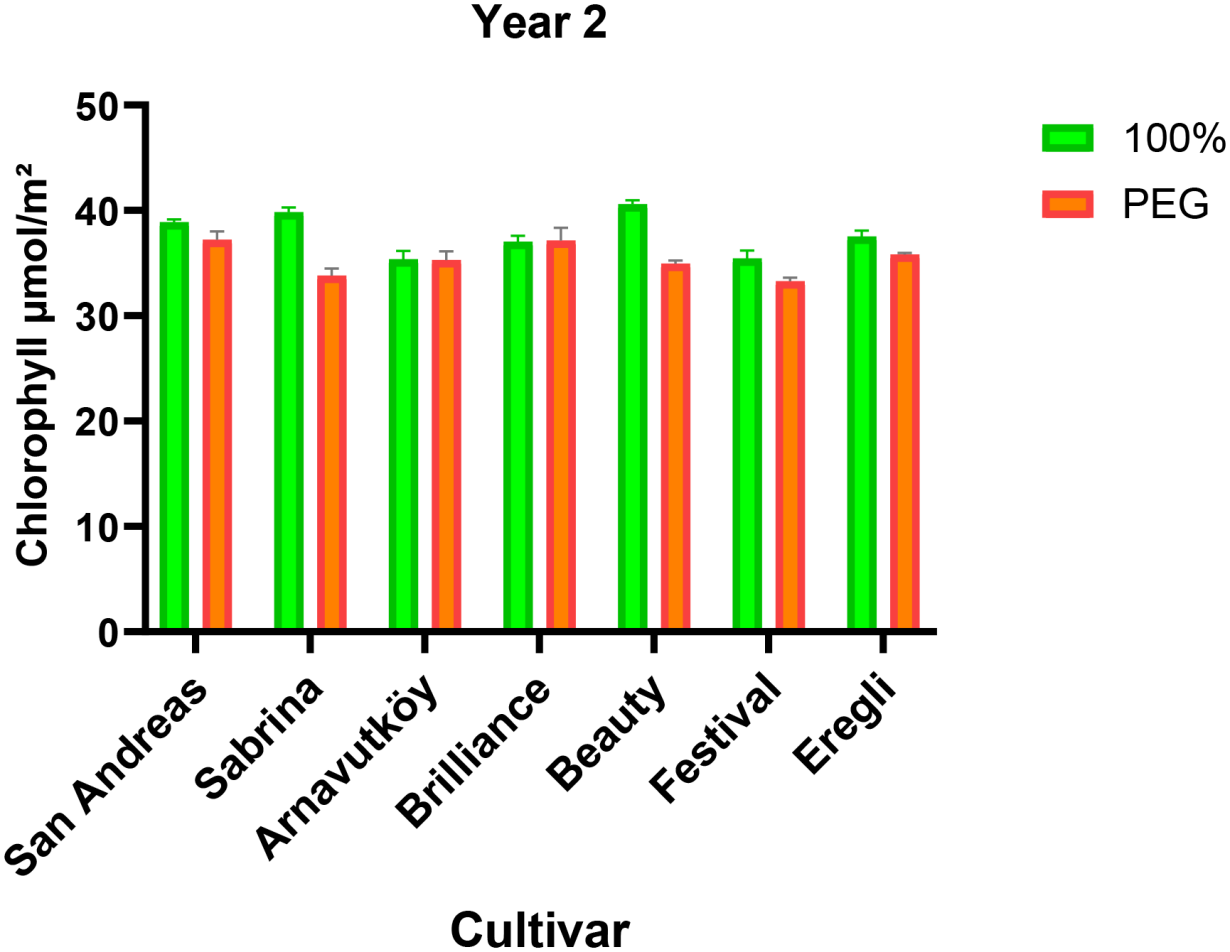
Chlorophyll content (μmol m^-2^) of strawberry cultivars under control and PEG-induced drought stress in Year 2 (2024).

The visual representation of these changes for the first year is provided in **Figure 1**, which highlights the cultivar-specific declines in chlorophyll content under drought conditions. Likewise, changes observed in the second year are illustrated in **Figure 2**, further emphasizing the seasonal differences and variation among genotypes.

The observed pigment loss is consistent with the findings of Zahedi et al. (2023), who reported that drought stress in strawberry leads to a reduction in chlorophyll and carotenoid levels as part of a photoprotective mechanism. This physiological adjustment helps minimize light absorption and protects the photosynthetic apparatus from photooxidative damage when water is limited.

Notably, baseline chlorophyll values in the second year were significantly lower than those in the first—an overall 26.7% decrease in control plants—likely due to elevated temperatures and higher light intensity during the summer season.

#### 3.2.3 Relative water content

Relative water content (RWC) declined significantly under drought stress in all cultivars across both years (P< 0.001), indicating a clear physiological response to water limitation. In Year 1, mean RWC dropped from 77.9% to 67.7% (a 13.1% reduction), and in Year 2, from 93.0% to 86.0% (a 7.5% reduction). These results, presented in **Table 1** (Year 1) and **Table 2** (Year 2), confirm RWC as a reliable and sensitive indicator of plant water status under drought conditions. This trend is consistent with the findings of Zahedi et al. (2023), who also reported simultaneous reductions in chlorophyll and carotenoid contents under water deficit.

Maintaining higher RWC under drought is widely recognized as a key trait associated with drought tolerance. As shown in **Tables 1**,**2**, all cultivars experienced reductions in RWC, though second-year plants generally retained more water under both control and stress conditions. This improved water retention may reflect enhanced root development or improved hydraulic functioning over time. Gonzalez-Fuentes et al. (2016) emphasized the contribution of stable root zone temperature in supporting water uptake and RWC maintenance in strawberries.

Among the tested cultivars, Brilliance exhibited the highest RWC under drought stress in both years (67.9% and 89.2%, respectively), indicating superior water retention capacity. In contrast, Sabrina experienced the greatest RWC decline in Year 1 (25.1%), but showed notable improvement in Year 2 (9.7%), suggesting possible physiological acclimation or adaptive adjustments.

**Figure 3:**
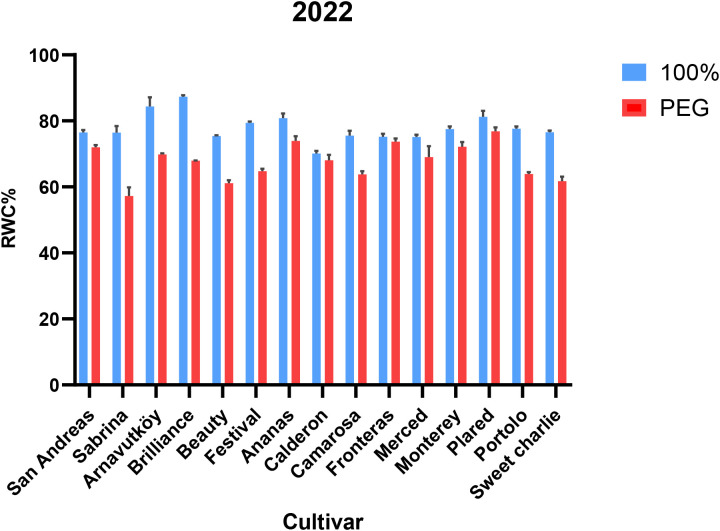
Relative water content (RWC, %) of strawberry cultivars under control and PEG-induced drought stress in Year 1 (2022).

**Figure 4:**
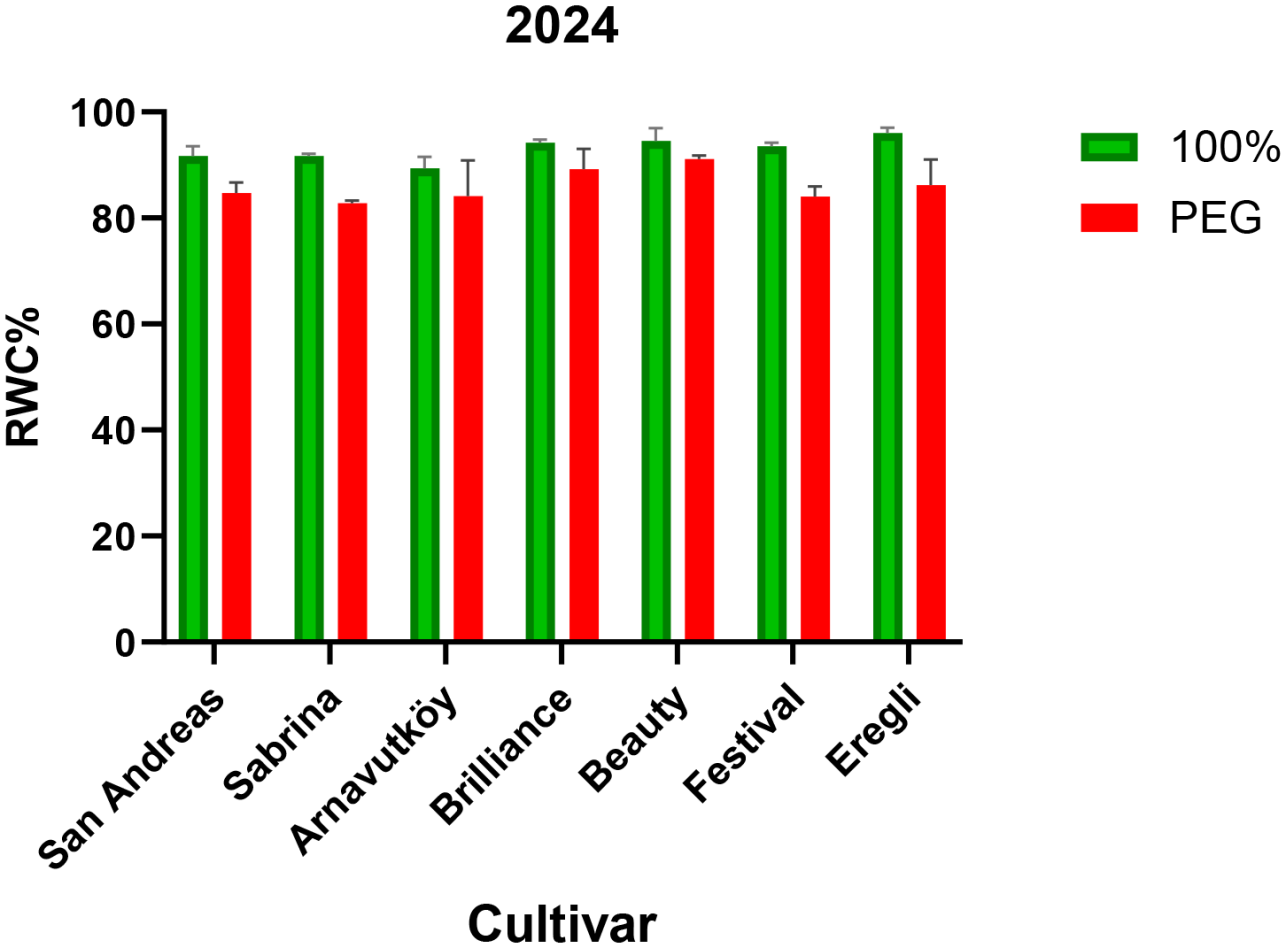
Relative water content (RWC, %) of strawberry cultivars under control and PEG-induced drought stress in Year 2 (2024).

These cultivar-specific responses are visually illustrated in **Figure 3** (Year 1) and **Figure 4** (Year 2), highlighting the contrasting water retention behaviors under PEG-induced drought stress. Further research may help elucidate the physiological or molecular mechanisms underlying the improved drought resilience observed in cultivars such as Sabrina.

#### 3.2.4 Photosynthetic quantum yield

Photosystem II (PSII) efficiency significantly declined under drought stress in most strawberry cultivars (P < 0.01). In the first year, mean values decreased from 0.63 to 0.58 (a 7.9% reduction), while in the second year they dropped from 0.74 to 0.65 (a 12.2% decline). These results, presented in **Table 1** and **2**, confirm that drought stress adversely affects PSII function across both growing season .Such findings are consistent with previous studies reporting that water deficit disrupts the electron transport chain in PSII, leading to diminished photosynthetic performance. For instance, Razavi et al. (2008) demonstrated that drought stress significantly reduces the efficiency of energy capture by PSII in strawberry. This physiological impairment is closely associated with reduced plant growth and yield under water-limited conditions.

Among the tested cultivars, Brilliance maintained the highest PSII efficiency under drought in the second year (0.75), with only a 2.6% reduction compared to the control. This suggests the presence of effective photoprotective mechanisms and stable PSII performance under stress, supporting its classification as a drought-tolerant genotype. Arief et al. (2023) also emphasized the value of chlorophyll fluorescence imaging as a non-invasive and rapid tool for detecting early drought and heat stress symptoms in strawberry, reinforcing the role of PSII-related parameters as reliable physiological markers in screening for stress-tolerant cultivars.

The stable photosynthetic efficiency observed in Brilliance highlights its potential for use in breeding programs targeting drought resilience and supports the application of fluorescence-based phenotyping tools in cultivar selection.

#### 3.2.5 Leaf dry matter content

Leaf dry matter content (LDMC) increased significantly in all cultivars under drought stress during both experimental years (P< 0.001), reflecting a common adaptive response to water limitation. In Year 1, LDMC rose from 29.4% to 34.0% (a 15.6% increase), and in Year 2 from 26.3% to 30.7% (a 16.7% increase). These data, summarized in **Table 1** (Year 1) and **Table 2** (Year 2), indicate a shift in carbon allocation toward structural compounds that enhance tissue rigidity and help maintain physiological function under dehydration.

The observed increase in LDMC is commonly associated with osmotic adjustment, as a higher proportion of dry matter relative to fresh weight supports cell wall stability and turgor maintenance. Ödemiş et al. (2020) emphasized the roles of osmotic regulation, reduced transpiration, and smaller leaf area in enhancing drought resilience in strawberries.

Among the tested cultivars, Plared exhibited the highest LDMC increase in Year 1 (35.1%), while Festival ranked highest in Year 2 (30.1%), suggesting effective osmotic regulation and drought-induced carbon partitioning. These changes may involve the accumulation of compatible solutes such as proline and soluble sugars, which protect cellular structures and sustain metabolic activity under stress. This interpretation is consistent with the findings of Save et al. (1993), who highlighted the importance of osmotic and elastic adjustments in strawberry leaves under mild water stress.

#### 3.2.6 Leaf number

Leaf number decreased significantly under drought stress in all cultivars across both years (P< 0.001), indicating a morphological drought avoidance strategy aimed at reducing transpirational surface area. In Year 1, the average number of leaves dropped from 17.9 to 10.8 (a 39.7% reduction), and in Year 2, from 15.6 to 5.8 (a 62.8% reduction). These data are presented in **Tables 1**, **2**, which summarize cultivar-specific changes in leaf number under both irrigation regimes.

This reduction likely reflects both decreased leaf initiation and accelerated senescence. Ödemiş et al. (2020) reported similar findings, noting that increased drought severity reduces both leaf number and leaf area in strawberries—traits associated with reduced light interception and water use. The sharper decline observed in Year 2 may suggest a shift in resource allocation toward reproductive organs in physiologically more mature plants.

Nezhadahmadi et al. (2015) also observed significant reductions in leaf number and area across strawberry cultivars under water stress, with genotype-dependent variability. In our study, Arnavutköy exhibited the least reduction in Year 1 (22.9%), indicating better vegetative maintenance under stress, while Calderon showed the largest decline (59.1%), suggesting greater sensitivity. Similarly, Ghaderi et al. (2015) found that cultivar-specific responses to drought could be alleviated by exogenous application of salicylic acid, which helped maintain leaf number under water-limited conditions.

#### 3.2.7 Carbohydrate accumulation dynamics

The analysis of carbohydrate content in strawberry leaves revealed significant differences in drought response mechanisms between the first and second years of cultivation. In the first year, all cultivars exhibited a marked increase in total sugar content under PEG-induced drought stress (**Table 3**). The most notable response was observed in the Sweet Charlie cultivar, where total sugar content rose from 3.48% under control conditions to 28.42% under drought (an 8.2-fold increase). Similarly, Monterey and Fronteras showed sharp increases, from 2.69% to 22.90% (8.5-fold) and 13.58% to 25.43% (1.9-fold), respectively. This pronounced carbohydrate accumulation reflects a classic osmotic adjustment mechanism, whereby plants synthesize and store compatible solutes to lower osmotic potential and maintain water uptake during water deficit. These findings are consistent with those of Zahedi et al. (2023), who reported enhanced soluble sugar accumulation under drought stress in strawberry. According to their study, this response plays a key role in maintaining cell turgor and protecting cellular structures during dehydration.

**Table 3:** Biochemical traits of strawberry cultivars under drought stress (PEG) and well-watered conditions in the first year.

Cultivar	Dried leaf sucrose content (%)		Dried leaf glucose content (%)		Dried leaf fructose content (%)		Dried leaf xylose content (%)		Dried leaf total sugar (%)		Antioxidant activity (%)		Free radical scavenging (%)		Dried leaf succinic acid content (%)	
	100%	PEG	100%	PEG	100%	PEG	100%	PEG	100%	PEG	100%	PEG	100%	PEG	100%	PEG
Ananas	1.91 ± 0.08 i	4.04 ± 0.07 f	5.65 ± 0.30 gh	7.71 ± 0.04 cd	6.21 ± 0.30 gh	7.41 ± 0.04 e	0.31 ± 0.07 gh	0.36 ± 0.04 fg	14.08 ± 0.45 l	19.51 ± 0.05 f	75.68 ± 0.25 kl	78.58 ± 0.06 e	88.66 ± 0.14 ef	91.58 ± 0.07 a	16.98 ± 0.80 f	17.31 ± 0.39 ef
Arnavutköy	0.99 ± 0.02 kl	4.16 ± 0.03 f	4.91 ± 0.01 i	6.23 ± 0.17 f	5.70 ± 0.01 jk	6.24 ± 0.24 gh	0.19 ± 0.01 i	0.28 ± 0.03 h	11.78 ± 0.02 n	16.91 ± 0.41 j	75.58 ± 0.25 kl	76.47 ± 0.38 hi	87.28 ± 0.24 h	91.55 ± 0.52 a	5.61 ± 0.05 op	9.98 ± 0.20 l
Beauty	1.51 ± 0.03 ij	7.33 ± 0.01 c	1.63 ± 0.07 k	5.74 ± 0.02 g	1.53 ± 0.10 no	5.46 ± 0.11 jkl	0.18 ± 0.02 i	0.42 ± 0.01 cde	4.86 ± 0.15 p	18.95 ± 0.09 g	87.39 ± 0.27 d	91.41 ± 0.07 a	75.61 ± 0.25 l	77.92 ± 0.06 i	10.59 ± 0.01 k	15.04 ± 0.04 h
Brilliance	1.64 ± 0.01 i	3.45 ± 0.02 gh	4.37 ± 0.06 j	6.86 ± 0.13 e	4.52 ± 0.06 m	6.92 ± 0.10 f	0.28 ± 0.01 h	0.31 ± 0.01 gh	10.80 ± 0.12 o	17.53 ± 0.23 i	.	.	.	.	15.79 ± 0.40 g	18.55 ± 0.01 d
Camarosa	1.53 ± 0.03 ij	5.11 ± 1.08 e	4.86 ± 0.02 i	6.03 ± 0.47 fg	5.02 ± 0.01 l	6.29 ± 0.64 gh	0.36 ± 0.02 fg	0.45 ± 0.01 bcd	11.76 ± 0.07 n	17.88 ± 0.31 hi	75.52 ± 0.21 kl	77.24 ± 0.19 g	87.81 ± 0.20 g	90.73 ± 0.19 bc	23.74 ± 0.25 a	12.54 ± 0.02 j
Ereğli	0.78 ± 0.30 klm	8.20 ± 0.19 b	1.52 ± 0.56 kl	8.54 ± 0.01 b	1.63 ± 0.60 n	8.36 ± 0.13 b	0.09 ± 0.02 jk	0.29 ± 0.10 h	3.53 ± 0.37 r	25.39 ± 0.05 b	.	.	.	.	7.44 ± 0.49 n	17.57 ± 0.20 ef
Festival	1.06 ± 0.26 jk	5.31 ± 0.03 e	1.53 ± 0.39 kl	6.15 ± 0.10 f	1.40 ± 0.44 no	6.13 ± 0.13 hi	0.17 ± 0.05 i	0.35 ± 0.05 fg	4.17 ± 0.45 q	17.94 ± 0.25 h	88.36 ± 0.04 c	90.70 ± 0.22 b	76.06 ± 0.05 k	77.21 ± 0.23 j	6.01 ± 0.60 o	12.24 ± 0.53 j
Fronteras	3.29 ± 0.04 h	9.11 ± 0.12 a	4.55 ± 0.01 ij	7.86 ± 0.13 cd	5.26 ± 0.06 kl	7.97 ± 0.14 bc	0.48 ± 0.03 b	0.49 ± 0.05 b	13.58 ± 0.06 m	25.43 ± 0.10 b	75.29 ± 0.19 l	77.60 ± 0.18 fg	88.78 ± 0.17 ef	90.61 ± 0.18 c	16.01 ± 0.05 g	19.13 ± 0.09 cd
Merced	3.10 ± 0.10 h	4.85 ± 0.08 e	4.67 ± 0.02 ij	7.98 ± 0.10 c	5.40 ± 0.07 jkl	7.85 ± 0.04 cd	0.42 ± 0.00 cde	0.37 ± 0.03 efg	13.58 ± 0.18 m	21.04 ± 0.16 e	76.39 ± 0.06 hi	75.75 ± 0.04 jk	88.88 ± 0.11 de	88.64 ± 0.17 ef	20.07 ± 0.26 b	17.93 ± 0.05 e
Monterey	0.38 ± 0.08 m	6.16 ± 0.05 d	0.84 ± 0.08 no	8.01 ± 0.05 c	1.36 ± 0.29 no	8.27 ± 0.07 bc	0.04 ± 0.02 kl	0.47 ± 0.06 bc	2.69 ± 0.21 t	22.90 ± 0.13 c	76.64 ± 0.27 h	77.62 ± 0.39 fg	89.21 ± 0.27 d	90.63 ± 0.39 c	3.94 ± 0.25 r	14.94 ± 0.12 h
Plared	0.54 ± 0.01 klm	7.58 ± 0.17 c	0.73 ± 0.02 o	5.85 ± 0.06 fg	0.84 ± 0.01 p	5.75 ± 0.07 ij	0.07 ± 0.01 jkl	0.37 ± 0.02 efg	2.17 ± 0.04 u	19.54 ± 0.02 f	74.62 ± 0.08 m	78.82 ± 0.11 e	87.19 ± 0.08 h	91.83 ± 0.11 a	5.29 ± 0.11 p	19.36 ± 0.05 c
Portolo	0.48 ± 0.01 lm	3.87 ± 0.08 fg	1.16 ± 0.11 lmn	7.03 ± 0.06 e	1.29 ± 0.06 no	6.65 ± 0.08 fg	0.05 ± 0.00 jkl	0.35 ± 0.01 fg	2.99 ± 0.19 st	17.90 ± 0.06 hi	75.81 ± 0.05 jk	77.78 ± 0.21 f	88.38 ± 0.05 f	90.78 ± 0.21 bc	4.63 ± 0.33 q	13.43 ± 0.20 i
Sabrina	0.65 ± 0.01 klm	3.80 ± 1.29 fg	0.88 ± 0.11 no	5.33 ± 0.70 h	1.07 ± 0.13 op	5.57 ± 0.74 jk	0.11 ± 0.01 j	0.25 ± 0.03 h	2.70 ± 0.23 t	14.45 ± 0.48 k	76.10 ± 0.26 ij	77.64 ± 0.05 fg	88.59 ± 0.18 ef	91.10 ± 0.08 b	8.68 ± 1.07 m	9.87 ± 0.07 l
San Andreas	0.74 ± 0.06 klm	6.06 ± 0.03 d	1.10 ± 0.01 mno	7.55 ± 0.18 d	1.26 ± 0.05 nop	7.48 ± 0.12 de	0.02 ± 0.00 l	0.40 ± 0.02 def	3.11 ± 0.01 s	21.49 ± 0.29 d	74.78 ± 0.06 m	78.72 ± 0.64 e	87.35 ± 0.06 h	91.73 ± 0.63 a	5.51 ± 0.51 op	16.17 ± 0.15 g
Sweet charlie	0.60 ± 0.06 klm	6.28 ± 0.12 d	1.29 ± 0.09 klm	10.51 ± 0.02 a	1.55 ± 0.01 n	11.03 ± 0.04 a	0.05 ± 0.01 jkl	0.61 ± 0.05 a	3.48 ± 0.04 r	28.42 ± 0.11 a	75.56 ± 0.08 kl	78.47 ± 0.31 e	87.86 ± 0.09 g	91.96 ± 0.31 a	7.86 ± 0.26 n	17.37 ± 0.25 ef

Values followed by different lowercase letters within a column are significantly different at p< 0.05 (Duncan’s test).

**Figure 5:**
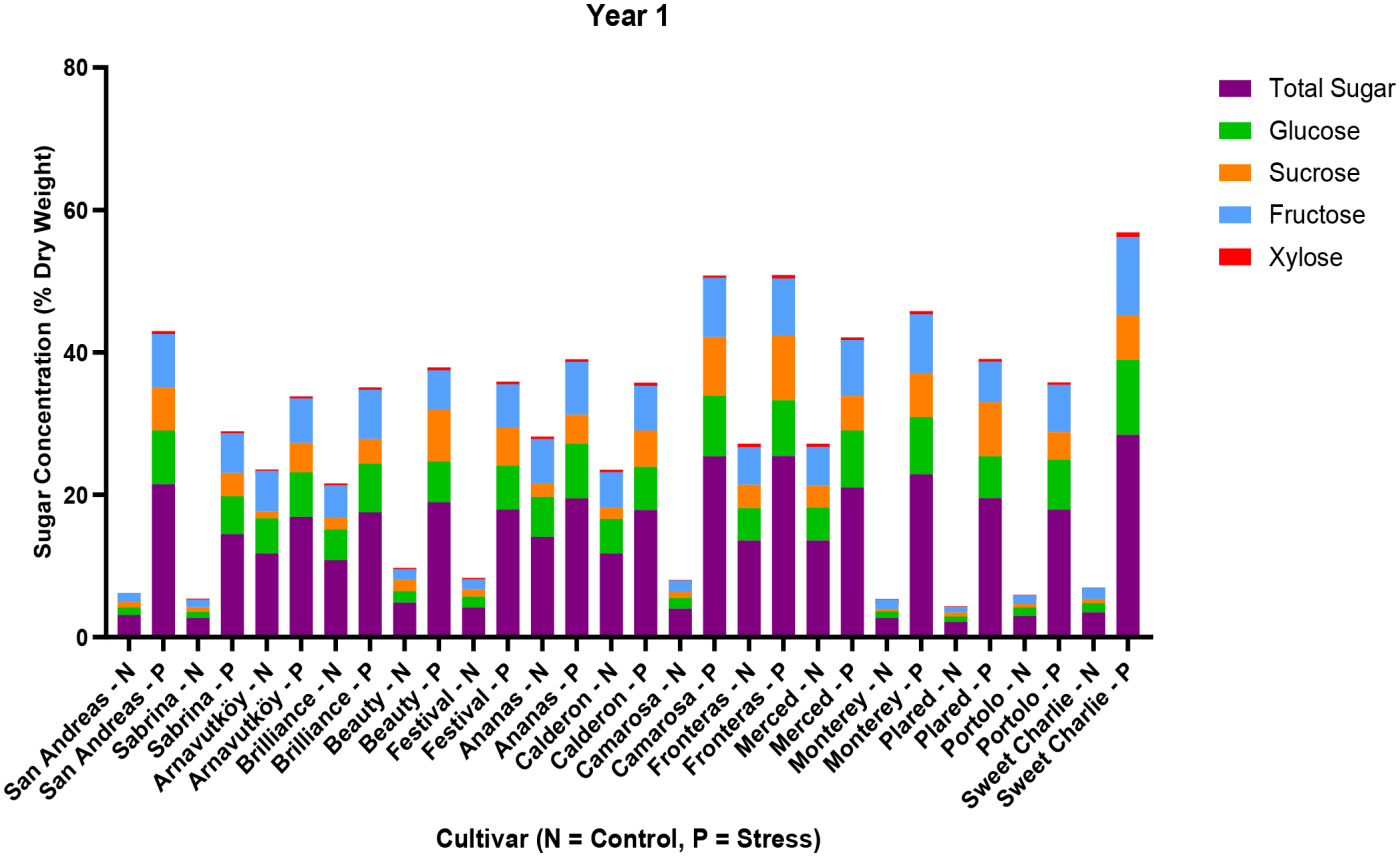
Sugar composition (sucrose, glucose, fructose, xylose) and total sugar content in leaf tissues of 15 strawberry cultivars under control (N) and PEG-induced drought stress in Year 1 (2022).

As illustrated in **Figure 5**, not only did total sugar content increase, but also the proportions of major sugars—glucose, fructose, and sucrose—shifted significantly under drought stress in Year 1. Glucose and fructose, in particular, exhibited the most substantial increases, with maximum values reaching 10.51% and 11.03% in Sweet Charlie, respectively. The relative contribution of each sugar varied by genotype, suggesting cultivar-specific metabolic adjustments in response to water deficit.

In contrast, the carbohydrate accumulation pattern changed considerably in the second year. As shown in **Table 4**, baseline sugar content in control plants was generally higher than in the first year (ranging from 3.00% to 7.48%), while the drought-induced increases were markedly reduced. For example, in San Andreas, total sugar content rose only slightly from 6.29% to 6.79% under drought—a mere 7.9% increase—compared to the 6.9-fold rise in the first year (from 3.11% to 21.49%). A similar pattern was observed in Beauty, which showed just a 2.2% increase in Year 2 (from 7.18% to 7.34%) versus a 3.9-fold increase in Year 1 (from 4.86% to 18.95%).

**Table 4:** Biochemical traits of strawberry cultivars under drought stress (PEG) and well-watered conditions in the second year.

Cultivar	Dried leaf sucrose content (%)		Dried leaf glucose content (%)		Dried leaf fructose content (%)		Dried leaf xylose content (%)		Dried leaf total sugar (%)		Antioxidant Activity (%)		Free radical scavenging (%)		Dried leaf succinic acid content (%)	
	100%	PEG	100%	PEG	100%	PEG	100%	PEG	100%	PEG	100%	PEG	100%	PEG	100%	PEG
Arnavutköy	1.00 ± 0.31 ef	1.04 ± 0.04 ef	2.39 ± 0.31 def	2.13 ± 0.02 efg	2.34 ± 0.33 cd	2.78 ± 0.11 b	0.23 ± 0.12 cde	0.18 ± 0.02 defg	5.96 ± 0.92 gh	6.13 ± 0.12 fgh	88.92 ± 0.25 f	89.17 ± 0.06 ef	81.54 ± 0.06 g	82.26 ± 0.25 ef	1.18 ± 0.39 g	2.21 ± 0.31 f
Beauty	0.65 ± 0.01 g	1.74 ± 0.08 c	3.06 ± 0.05 bc	2.67 ± 0.11 cd	3.33 ± 0.08 a	2.74 ± 0.03 b	0.15 ± 0.03 efg	0.19 ± 0.01 defg	7.18 ± 0.14 def	7.34 ± 0.18 cde	89.57 ± 0.30 cdef	90.37 ± 0.08 ab	81.94 ± 0.30 efg	83.70 ± 0.08 abc	2.29 ± 0.25 f	2.30 ± 0.09 f
Brilliance	1.21 ± 0.22 de	0.78 ± 0.03 fg	2.51 ± 0.43 de	3.70 ± 0.24 a	1.68 ± 0.25 e	3.49 ± 0.33 a	0.26 ± 0.11 cd	0.41 ± 0.08 a	5.65 ± 1.00 h	8.39 ± 0.46 abc	89.90 ± 0.10 bcd	90.07 ± 1.03 bc	82.43 ± 1.04 de	83.23 ± 0.10 bc	2.49 ± 0.34 f	2.57 ± 0.15 f
Ereğli	1.83 ± 0.20 c	1.65 ± 0.13 c	2.76 ± 0.08 cd	3.24 ± 0.04 b	2.77 ± 0.09 b	3.30 ± 0.04 a	0.12 ± 0.00 g	0.36 ± 0.01 ab	7.48 ± 0.37 bcd	8.54 ± 0.06 ab	89.74 ± 0.12 bcde	88.04 ± 0.63 g	83.07 ± 0.12 cd	80.41 ± 0.64 h	2.27 ± 0.08 f	3.15 ± 0.02 de
Festival	1.23 ± 0.45 de	3.10 ± 0.17 a	0.65 ± 0.08 h	2.85 ± 0.57 bcd	0.99 ± 0.20 f	2.56 ± 0.34 bc	0.13 ± 0.03 fg	0.30 ± 0.05 bc	3.00 ± 0.15 i	8.80 ± 1.11 a	89.34 ± 0.40 def	90.48 ± 0.07 ab	81.71 ± 0.39 fg	83.81 ± 0.07 ab	4.10 ± 0.17 c	4.65 ± 0.20 b
Sabrina	1.61 ± 0.02 c	1.52 ± 0.24 cd	2.03 ± 0.05 fg	2.76 ± 0.37 cd	2.48 ± 0.06 bc	3.14 ± 0.34 a	0.11 ± 0.00 g	0.22 ± 0.02 cdef	6.23 ± 0.12 fgh	7.65 ± 0.95 bcd	90.13 ± 0.35 bc	90.87 ± 0.01 a	82.49 ± 0.35 de	84.20 ± 0.01 a	2.95 ± 0.08 e	6.22 ± 0.12 a
San Andreas	2.24 ± 0.11 b	2.42 ± 0.16 b	1.82 ± 0.17 g	1.99 ± 0.16 fg	2.10 ± 0.19 d	2.21 ± 0.14 cd	0.14 ± 0.02 efg	0.17 ± 0.03 defg	6.29 ± 0.48 efgh	6.79 ± 0.48 defg	88.23 ± 0.44 g	90.19 ± 0.15 abc	80.60 ± 0.44 h	83.52 ± 0.15 abc	3.48 ± 0.07 d	6.17 ± 0.15 a

Values followed by different lowercase letters within a column are significantly different at p< 0.05 (Duncan’s test).

**Figure 6:**
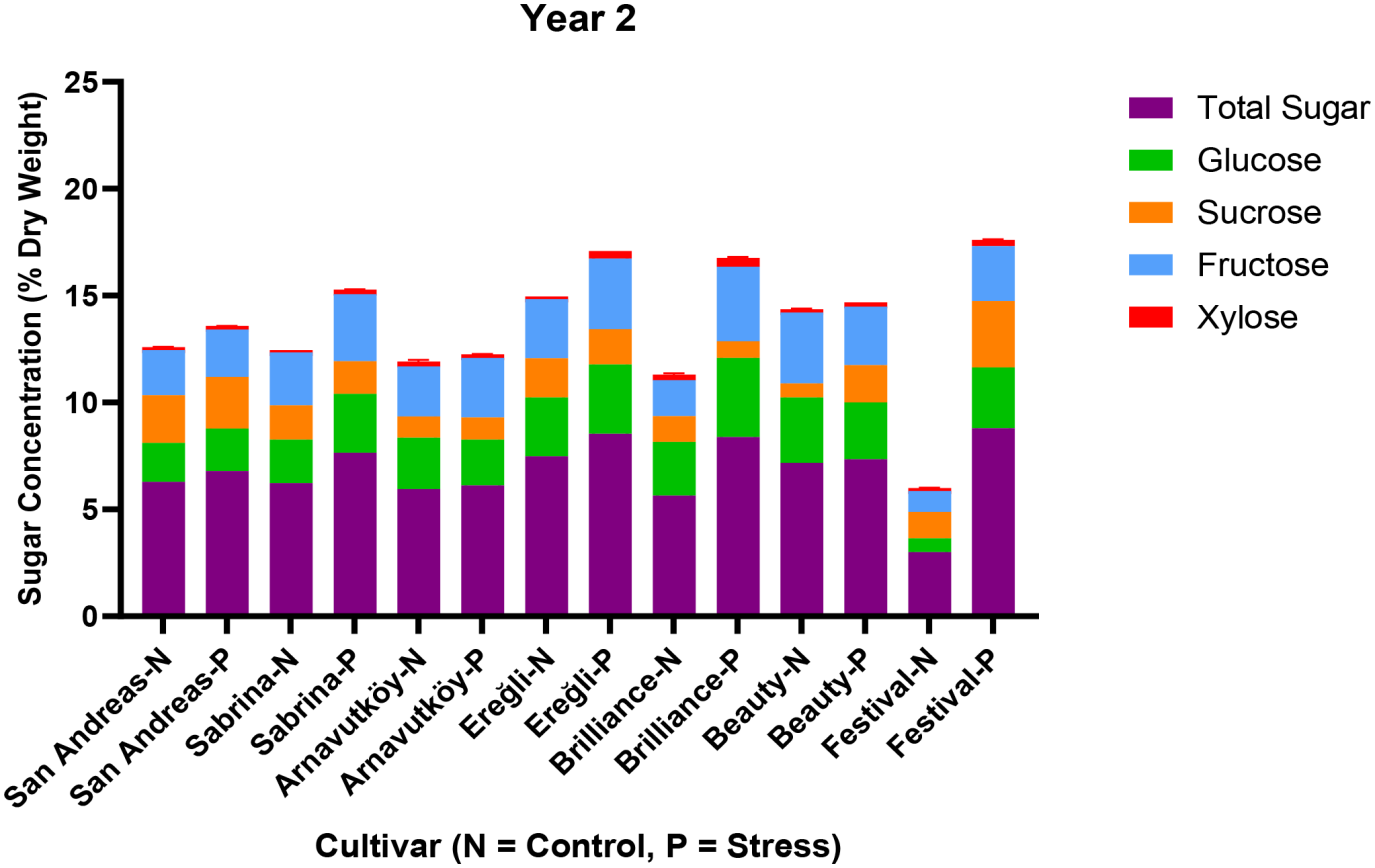
Sugar composition (sucrose, glucose, fructose, xylose) and total sugar content in leaf tissues of seven strawberry cultivars under control (N) and PEG-induced drought stress in Year 2 (2024).

**Figure 6** illustrates both total sugar content and individual sugar components under control and PEG-induced drought in the second year. Compared to Year 1, cultivars accumulated smaller amounts of glucose and fructose, and in some cases, sucrose concentrations remained nearly unchanged. This substantial shift in carbohydrate dynamics suggests a transition in drought response strategy between years. While the first-year responses are consistent with classical osmotic adjustment, the second-year data indicate the possible activation of alternative or complementary mechanisms, such as enhanced antioxidant activity, altered hormonal signaling, or improved membrane stability.

This interpretation is further supported by Ghaderi and Siosemardeh (2011), who reported that both soluble carbohydrates and proline contribute to osmotic adjustment in strawberry under drought conditions. However, when sugar accumulation is limited as observed in Year 2 other stress mitigation pathways may take precedence. Moreover, Sun et al. (2015) demonstrated that carbohydrate accumulation under drought often accompanies broader metabolic changes, including the activation of antioxidant enzymes that help protect against oxidative stress.

The observed differences in sugar composition and accumulation across years and cultivars highlight the dynamic and genotype-dependent nature of drought response in strawberry and emphasize the importance of multi-year evaluations for the reliable identification of drought-tolerant genotypes.

#### 3.2.8 Antioxidant capacity and free radical scavenging capacity

Drought stress consistently enhanced antioxidant activity across most strawberry cultivars, indicating the activation of both enzymatic and non-enzymatic defense systems against oxidative damage. In the first year, cultivar responses to PEG treatment varied considerably: Merced showed minimal change (from 76.39% under control to 75.75% under stress), whereas Beauty and Plared exhibited notable increases (from 87.39% to 91.41% and 74.62% to 78.82%, respectively), as presented in **Table 3**. In the second year, all seven tested cultivars showed elevated antioxidant levels under PEG-induced drought, with Festival demonstrating the highest activity (90.48%), closely followed by Sabrina (90.87%) and Beauty (90.37%) (**Table 4**). These findings indicate robust and stable antioxidant defense systems in these genotypes.

Strawberry plants under drought stress are known to upregulate both enzymatic antioxidants—such as superoxide dismutase (SOD), catalase (CAT), and peroxidases (POD)—and non-enzymatic compounds like flavonoids and polyphenols. These systems function synergistically to scavenge reactive oxygen species (ROS), thereby protecting lipids, proteins, and DNA from oxidative damage (Zahedi et al., 2020, 2023; Mishra et al., 2023).

The genotypic differences observed in our study highlight the varying capacities of cultivars to activate such protective mechanisms under water-deficit conditions, underscoring the importance of antioxidant-based screening in drought tolerance evaluation.

##### 3.2.8.1 Free radical scavenging capacity

Free radical scavenging activity also followed a consistent pattern of enhancement under drought stress across both years, underscoring its critical role in reactive oxygen species (ROS) detoxification. In the first year, all measured cultivars exhibited increased scavenging capacity under PEG treatment. The most substantial increases were observed in San Andreas (from 87.35% to 91.73%), Arnavutköy (87.28% to 91.55%), and Sweet Charlie (87.86% to 91.96%), as detailed in **Table 3**. Festival showed the lowest increase in scavenging capacity (from 88.14% to 89.29%), yet still exhibited a positive trend.

Second-year results were consistent, with elevated scavenging activity across all cultivars. Festival again recorded the highest value (83.81%), with Sabrina (84.20%) and Beauty (83.70%) showing similarly strong performance, as shown in **Table 4**. These findings suggest that efficient ROS scavenging is a reliable and widespread mechanism of drought tolerance in strawberry.

Previous studies have demonstrated that polyphenols and flavonoids accumulate under drought conditions and contribute to ROS neutralization and membrane stabilization (Nakabayashi et al., 2014; Rahimi et al., 2021). Our results support this concept: cultivars with stronger scavenging responses—such as San Andreas, Sweet Charlie, and Arnavutköy—may possess inherent biochemical traits associated with improved drought resilience.

### 3.3 Correlation analysis

Pearson’s correlation analysis was performed for strawberry cultivars evaluated under both well-watered (control) and PEG-induced drought stress conditions across two consecutive growing seasons (2022 and 2024).

**Figure 7:**
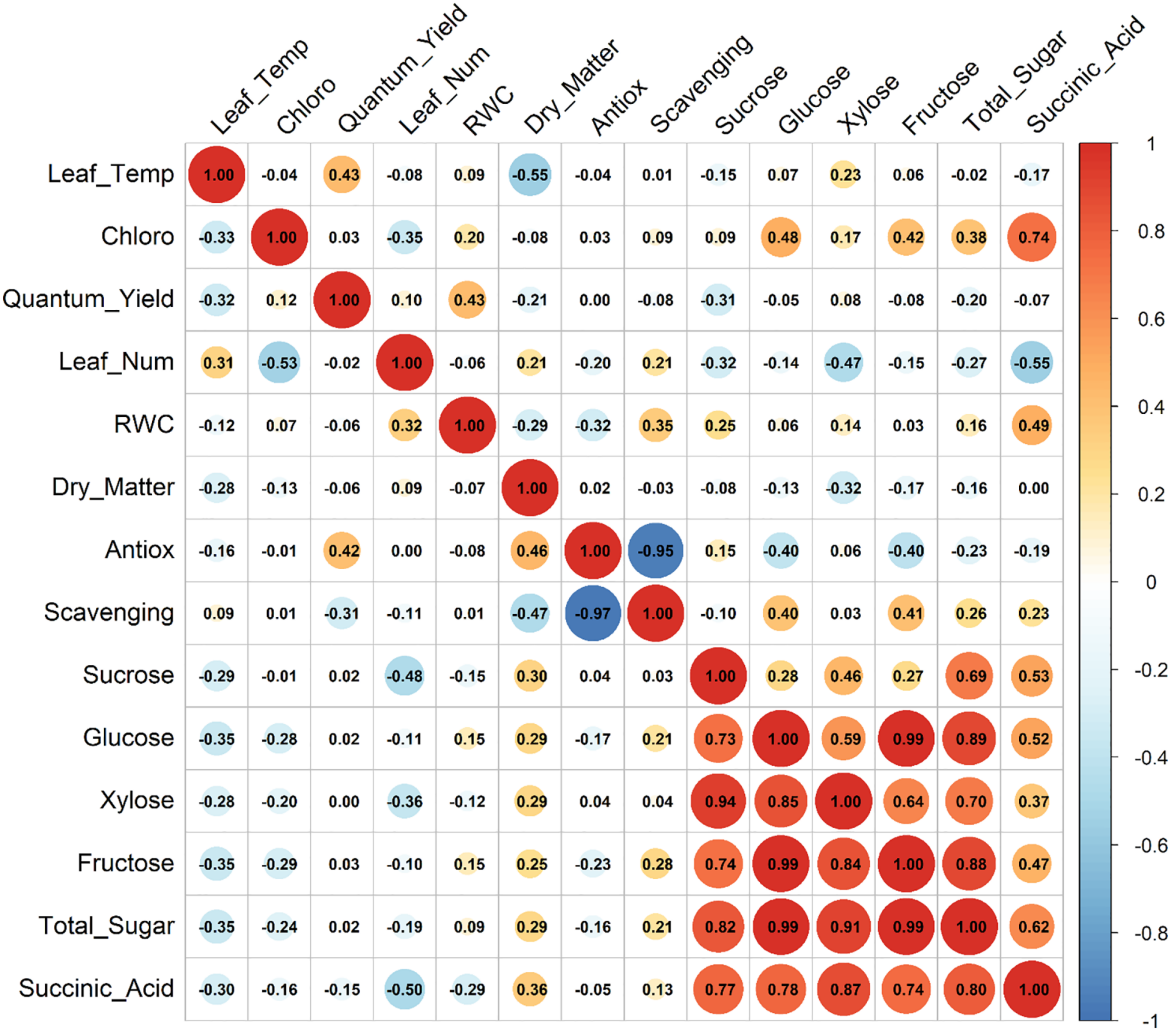
Correlation matrix of physiological, biochemical strawberry cultivars under drought (top) and control (bottom) conditions in Year 1 (2022).

Strong positive correlations among sugar components in the first year (**Figure 7**) highlight the coordinated regulation of carbohydrate metabolism under drought stress and underscore the central role of soluble sugars in osmotic adjustment. The near-perfect correlation between glucose and fructose (r = 0.99) under both control and stress conditions reflects their close metabolic linkage and interconversion, contributing jointly to the cellular osmolyte pool. The marked increase in the sucrose–fructose correlation from r = 0.74 under control to r = 0.99 under drought suggests a tighter metabolic regulation aimed at optimizing osmoprotectant accumulation. This observation aligns with Zahedi et al. (2023), who identified sugar accumulation as a key strategy for enhancing drought tolerance in strawberries. Such coordination likely supports more efficient osmotic potential reduction, promoting water uptake and turgor maintenance under limited water availability.

**Figure 8:**
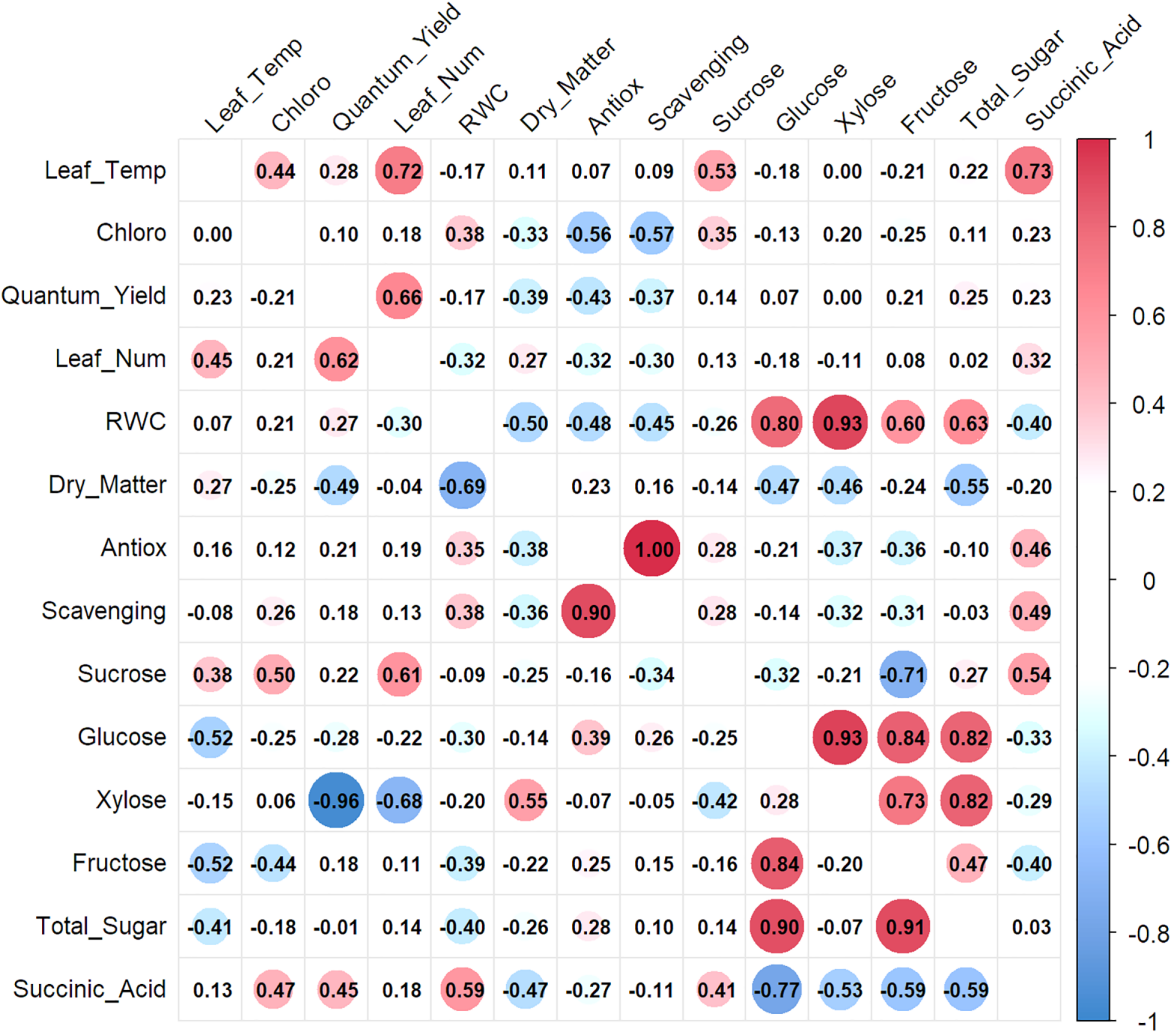
Correlation matrix of physiological, biochemical, in strawberry cultivars under drought (top) and control (bottom) conditions in Year 2 (2024).

In the second year, stronger correlations between relative water content (RWC) and sugars—including sucrose (r = 0.80), glucose (r = 0.93), and xylose (r = 0.60)—reflect a possible shift toward more effective osmotic regulation in more mature or acclimated plants. These findings, illustrated in **Figure 8**, support previous research by Zahedi et al. (2023), which demonstrated that drought-tolerant cultivars accumulate higher levels of osmoprotectants, helping sustain better RWC under stress. Thus, sugars not only serve key metabolic roles but may also act as indicators and regulators of water status during prolonged drought.

Correlations involving photosynthetic pigments further reveal nuanced drought effects on photosynthetic efficiency and metabolic coordination. In Year 1, the weakening of chlorophyll’s associations with sugars under stress indicates reduced photosynthetic capacity—a common drought symptom. In contrast, Year 2 presented a distinct pattern (**Figure 8**), where strong correlations were observed between leaf temperature, quantum yield, and succinic acid, suggesting enhanced cross-talk between thermal stress and mitochondrial adjustments under drought. These trends are consistent with Gao et al. (2024), who reported that increased leaf temperature and ABA levels under drought can influence photosystem II efficiency and mitochondrial respiration through ABA- and polyamine-mediated signaling pathways.

The antioxidant defense system also exhibited a tightly coordinated internal response, as evidenced by strong negative correlations between antioxidant capacity and scavenging activity. The near-perfect correlation observed in Year 2 under drought implies a more robust and possibly more efficient antioxidant response, potentially associated with plant maturity or repeated stress exposure. Sun et al. (2015) reported concurrent increases in antioxidant enzyme activity and soluble sugars in strawberry leaves under drought, indicating an integrated osmoprotective and antioxidative defense mechanism.

Overall, the year-to-year consistency in sugar-related metabolic networks, coupled with enhanced integration of water status, metabolic function, and thermal stress responses in Year 2, suggests the presence of cumulative acclimation. This metabolic plasticity may help strawberry cultivars better withstand prolonged drought stress by reinforcing osmotic balance and mitigating both thermal and oxidative damage. The persistent high correlations among sugars reaffirm their central role in drought resilience and suggest that enhancing carbohydrate metabolism could be a promising target in strawberry breeding programs for improved drought tolerance.

These correlation patterns provide valuable insight into the complex physiological and biochemical coordination underlying strawberry responses to water deficit. The interdependence of osmolyte accumulation, photosynthetic performance, and antioxidant defense mechanisms reflects an integrated response network that dynamically adjusts to sustain plant function under drought stress.

### 3.4 Principal component analysis

**Figure 9:**
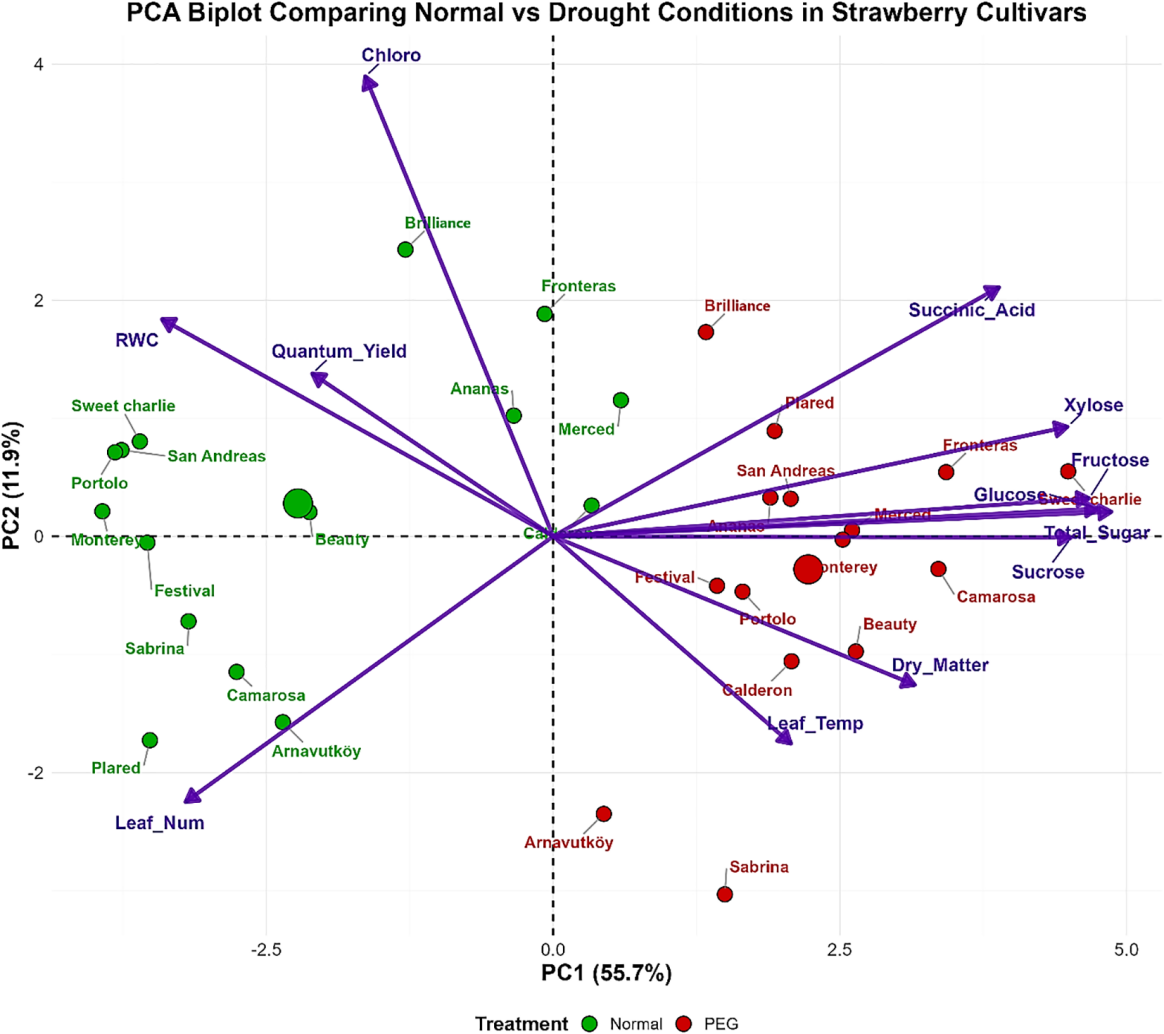
PCA biplot of strawberry cultivars based on physiological and biochemical traits under control (100% field capacity) and PEG-induced drought in 2022.

Principal component analysis (PCA) of the first-year data (**Figure 9**) effectively discriminated strawberry cultivars based on their drought stress responses, with PC1 and PC2 explaining 67.6% of total variance (PC1: 55.7%, PC2: 11.9%). The biplot demonstrated clear separation between normal and drought-stressed treatments, with cultivars showing systematic displacement from the left quadrants (normal conditions) to the right quadrants (drought stress), indicating comprehensive physiological reorganization under water deficit.

PC1 was dominated by sugar metabolism components including total sugar, sucrose, glucose, fructose, and xylose, all loading positively on this axis. This pattern establishes carbohydrate accumulation as the primary discriminating factor in drought responses, reflecting the critical role of osmotic adjustment in stress tolerance. Cultivars such as Monterey, Camarosa, and Beauty exhibited substantial positive displacement under drought stress, indicating pronounced metabolic adjustments, while Sweet Charlie, San Andreas, and Portolo maintained more conservative positions. This aligns with findings by Zahedi et al. (2023), who reported that sugars serve as osmoprotectants stabilizing cellular structures and maintaining turgor in strawberry under drought conditions. The variation among cultivars suggests genetic diversity in drought adaptation strategies, consistent with Ünal and Okatan (2023).

PC2 was characterized by photosynthetic parameters including chlorophyll content, leaf number, relative water content, and quantum yield, representing plant vigor and photosynthetic capacity. The negative association of succinic acid with these variables revealed an inverse relationship between organic acid accumulation and photosynthetic performance under stress conditions. This suggests that succinic acid accumulation may act as a metabolic adjustment or stress signal when photosynthesis is impaired, supporting Kiliç (2023), who highlighted the role of succinic acid in modulating antioxidant activities and osmotic balance under drought. Antioxidant parameters loaded moderately on both components, confirming their fundamental but intermediate role in the stress response network.

**Figure 10:**
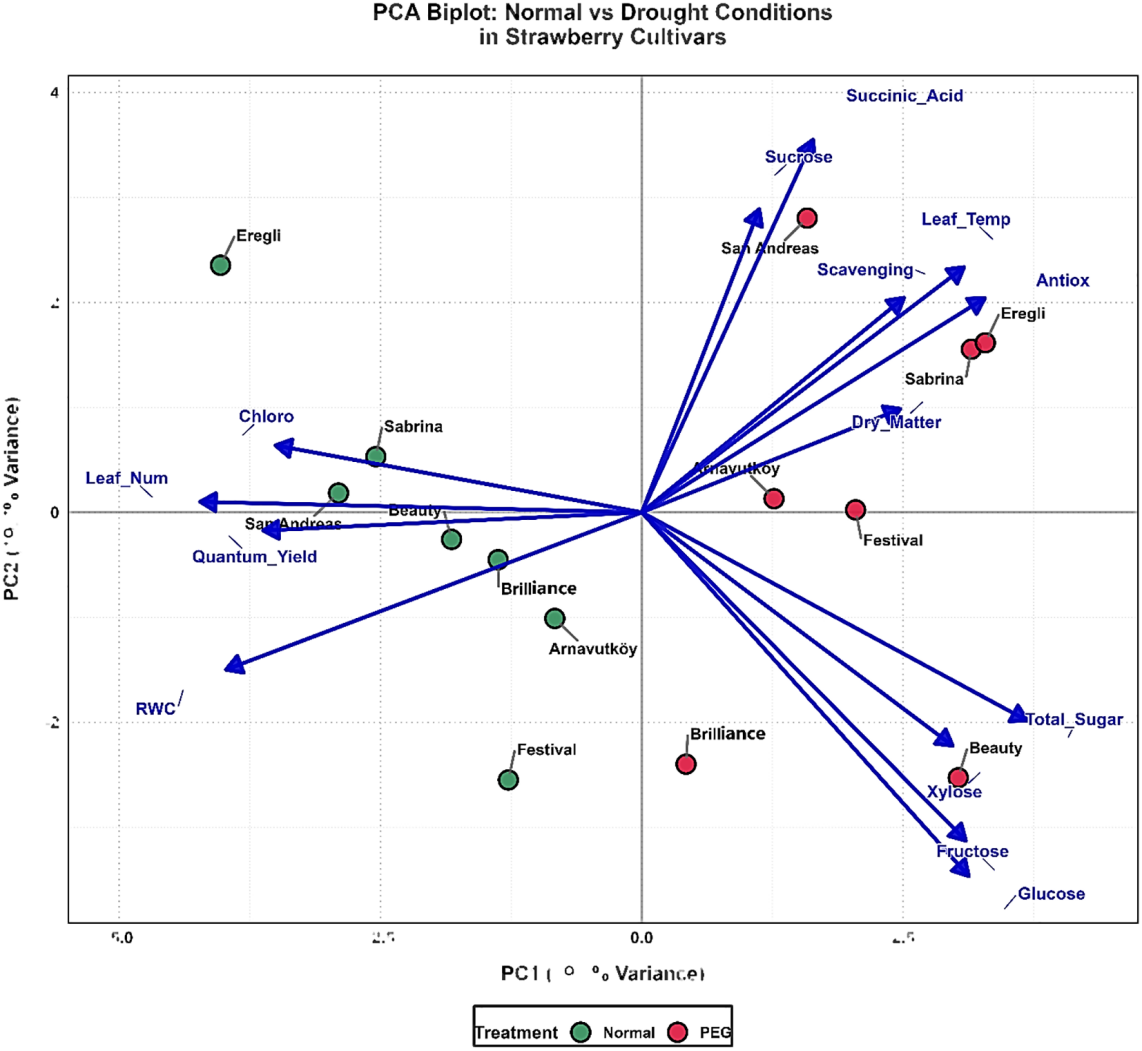
PCA biplot of strawberry cultivars based on physiological and biochemical traits under control (100% field capacity) and PEG-induced drought stress in 2024.

The second-year PCA analysis (**Figure 10**) of seven cultivars achieved enhanced treatment discrimination, with PC1 and PC2 explaining 58.0% of variance (PC1: 39.3%, PC2: 18.7%). The reduced cultivar number improved resolution of genotype-specific responses while maintaining clear separation between normal (blue clusters, left quadrants) and drought-stressed (yellow clusters, right side) treatments. PC1 remained dominated by carbohydrate metabolism variables, reinforcing sugar accumulation as a consistent drought tolerance mechanism. However, temporal variation in the importance of individual sugar components suggests environmental or developmental influences on metabolic responses, as noted by Mishra et al. (2023).

PC2 was characterized by succinic acid, showing the strongest positive loading, with antioxidant parameters contributing significantly. This indicates coordinated responses involving organic acid metabolism and antioxidant systems. Cultivar positioning revealed distinct drought tolerance phenotypes: Festival demonstrated stable responses, Arnavutköy and Brilliance balanced adaptation strategies, while Sabrina and San Andreas showed strong metabolic reorganization capacity under drought. The integration of leaf temperature and dry matter vectors in the biplot further highlighted morphophysiological adjustments complementing biochemical adaptations, reflecting the multifaceted nature of drought tolerance.

Cross-year comparison revealed consistent dominance of carbohydrate metabolism, validating sugar accumulation as a universal drought tolerance mechanism in strawberry. However, altered relative contributions of specific metabolites indicate that stress responses are modulated by environmental conditions, developmental stage, or genotype background. The enhanced treatment discrimination in the second year suggests that the selected cultivars represent distinct response phenotypes, offering clearer insight into tolerance mechanisms. The consistent positioning of antioxidant parameters across both years confirms their reliability as biochemical markers for drought stress evaluation, as supported by Şimşek (2024).

Overall, this PCA-based multidimensional analysis (**Figures 9**,**10**) captures coordinated adjustments across carbohydrate metabolism, photosynthetic capacity, organic acid accumulation, and antioxidant activity. It provides a comprehensive framework for identifying drought-tolerant strawberry genotypes and informs breeding strategies aimed at improving resilience amid increasing water scarcity.

### 3.5 Hierarchical cluster analysis

**Figure 11:**
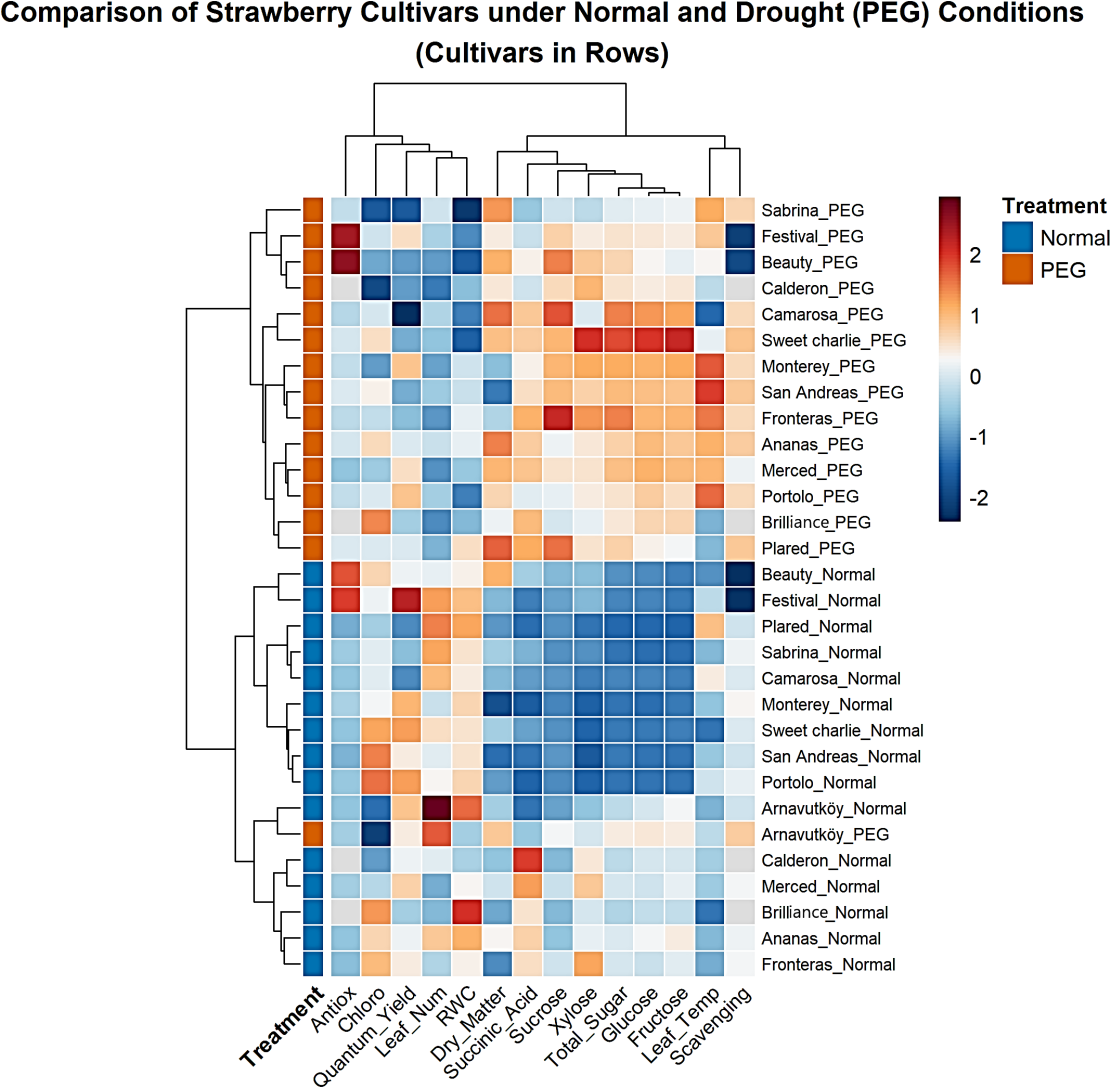
Clustered heatmap of 15 strawberry cultivars under control and PEG-induced drought stress conditions in Year 1 (2022).

**Figure 12:**
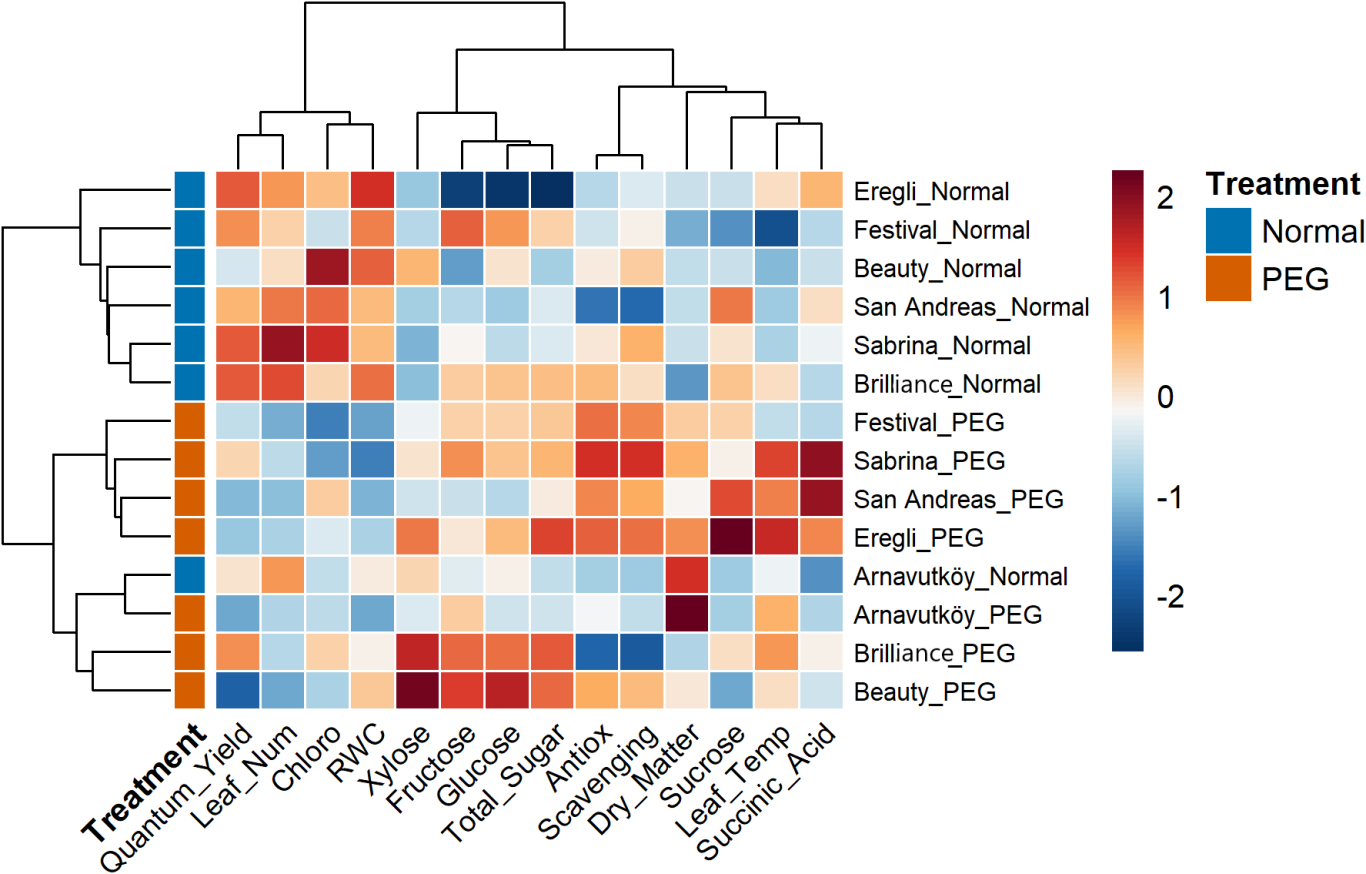
Clustered heatmap of 7 strawberry cultivars under control and PEG-induced drought stress conditions in Year 2 (2024).

Hierarchical cluster analysis of strawberry cultivars under PEG-induced drought stress revealed clear and consistent patterns across both experimental years. In **Figure 11** (Year 1) and **Figure 12** (Year 2), PEG-treated samples formed distinct clusters separated from control treatments. This clear treatment-based segregation reflects substantial and coordinated physiological and biochemical changes caused by drought stress and confirms the effectiveness of PEG as a reliable method for simulating water deficit conditions (Zahedi et al., 2023; Ödemiş et al., 2020).

In addition to treatment, separation, the analysis highlighted marked genetic differences in drought response strategies. Cultivars such as Sabrina, Festival, Beauty, Calderon, and Camarosa clustered separately under stress, suggesting distinct adaptive mechanisms. In contrast, Arnavutköy appeared in an intermediate position between clusters, possibly reflecting a moderate or mixed response. These clustering patterns are consistent with previous studies that report wide variation among strawberry genotypes in traits related to drought tolerance, such as sugar accumulation and photosynthetic performance (Ghaderi and Siosemardeh, 2011; Sun et al., 2015).

The heatmap color gradients in **Figures 11**, **12** clearly illustrate the regulation patterns of sugar components and chlorophyll. Traits such as total sugar, glucose, and fructose showed warm colors (red to orange) under PEG treatment in many cultivars, especially in Year 1, indicating strong upregulation likely as part of osmotic adjustment mechanisms. In contrast, chlorophyll content in some stressed cultivars displayed cooler tones (blue to light green), suggesting possible reductions and corresponding impacts on photosynthetic efficiency.

Interestingly, cultivars assessed in both years—such as Sweet Charlie and San Andreas—exhibited consistent clustering behavior, confirming that their responses were predominantly genotype-driven rather than environmentally dependent. Dendrogram branch lengths further reflected the intensity of stress responses: some cultivars shifted dramatically from their controls, indicating strong metabolic reprogramming, while others showed closer proximity to controls, suggesting milder responses (Ghaderi and Siosemardeh, 2011).

The clustering of sugar-related biochemical traits indicates that drought tolerance in strawberry is the result of coordinated metabolic adjustments, rather than isolated changes in individual traits. The simultaneous modulation of key carbohydrate markers—especially evident in Year 1—reflects a systematic physiological adaptation to drought stress, as also highlighted in recent metabolomic studies on perennial crops (Gao et al., 2024).

## 4 Conclusion

This study presents a comprehensive physiological and biochemical assessment of drought stress responses in a diverse set of Fragaria × ananassa cultivars under controlled PEG-induced water deficit conditions. Across two growing seasons, drought stress consistently impaired critical physiological functions, including photosynthetic efficiency, relative water content, and chlorophyll stability, while enhancing osmotic adjustment and antioxidant activity. The observed genotypic variation underscores the complex interplay between stress-induced damage and adaptive resilience mechanisms in strawberry. Sugar accumulation, particularly of glucose and fructose, emerged as a key drought tolerance strategy in the first year, reflecting strong osmotic adjustment. However, in the second year, this pattern shifted toward more subtle carbohydrate responses, suggesting either acclimation or a transition to alternative metabolic strategies such as enhanced ROS scavenging or membrane stabilization. Cultivars such as Sweet Charlie, Brilliance, San Andreas, and the local genotype Arnavutköy demonstrated robust biochemical and physiological resilience, evidenced by high antioxidant capacity, stable Fv/Fm ratios, and relatively maintained water content under drought conditions. The multivariate analyses (PCA and hierarchical clustering) further confirmed the genotypic specificity of drought responses and clearly delineated tolerant cultivars based on integrated trait performance. Notably, Brilliance maintained high photosynthetic quantum yield and RWC, while Festival and Beauty exhibited strong and stable antioxidant responses, marking them as promising candidates for drought-resilient breeding. In contrast, cultivars like Calderon and Plared exhibited more sensitive physiological profiles, indicating lower drought adaptability. Overall, the identification of cultivar-specific drought response patterns provides valuable insights for breeding programs aiming to enhance strawberry resilience under increasing water scarcity. Traits such as sugar partitioning, antioxidant potential, and photosystem II stability emerge as reliable markers for screening and selecting tolerant genotypes.

In addition to physiological and biochemical traits, future studies should also assess fruit yield and quality traits—such as fruit number, weight, Brix, and antioxidant content—to better reflect cultivar performance under drought. Integrating these with physiological responses can help identify reliable markers for breeding. While this study provides valuable insights, its limitations include the lack of a third year and environmental differences between the two seasons. Therefore, multi-year trials under both controlled and field conditions are recommended to ensure more robust and generalizable conclusions.

## Data Availability

The original contributions presented in the study are included in the article/**Supplementary Material**. Further inquiries can be directed to the corresponding author/s.

## References

[B1] Arief M. A. A. Kim H. Kurniawan H. Nugroho A. P. Kim T. Cho B. K. (2023). Chlorophyll fluorescence imaging for early detection of drought and heat stress in strawberry plants. Plants 12, 1387. doi: 10.3390/plants12061387, PMID: 36987075 PMC10057166

[B2] Ashraf M. Harris P. J. C. (2004). Potential biochemical indicators of salinity tolerance in plants. Plant Sci. 166, 3–16. doi: 10.1016/j.plantsci.2003.10.024

[B3] Baker N. R. (2008). Chlorophyll fluorescence: A probe of photosynthesis in *vivo* . Annu. Rev. Plant Biol. 59, 89–113. doi: 10.1146/annurev.arplant.59.032607.092759, PMID: 18444897

[B4] Barai K. Calderwood L. Wallhead M. Vanhanen H. Hall B. Drummond F.. (2022). High variation in yield among wild blueberry genotypes: Can yield be predicted by leaf and stem functional traits? Agronomy 12, 617. doi: 10.3390/agronomy12030617

[B5] Cao Q. Huang L. Li J. Qu P. Tao P. Crabbe M. J. C.. (2022). Integrated transcriptome and methylome analyses reveal the molecular regulation of drought stress in wild strawberry (Fragaria nilgerrensis). BMC Plant Biol. 22, 613. doi: 10.1186/s12870-022-03943-3, PMID: 36575384 PMC9795625

[B6] Chaves M. M. Flexas J. Pinheiro C. (2009). Photosynthesis under drought and salt stress: Regulation mechanisms from whole plant to cell. Ann. Bot. 103, 551–560. doi: 10.1093/aob/mcn125, PMID: 18662937 PMC2707345

[B7] Chen H. Jiang J. G. (2010). Osmotic adjustment and plant adaptation to environmental changes related to drought and salinity. Environ. Rev. 18, 309–319. doi: 10.1139/A10-014

[B8] Cordoba-Novoa H. A. Pérez M. M. Cruz B. E. Flórez N. Magnitskiy S. Moreno L. P. (2021). Shading reduces water deficit in strawberry (Fragaria × ananassa Duch.) plants during vegetative growth. bioRxiv. doi: 10.1101/2021.08.01.454682

[B9] Dai A. (2013). Increasing drought under global warming in observations and models. Nat. Climate Change 3, 52–58. doi: 10.1038/nclimate1633

[B10] Fang Y. Xiong L. (2015). General mechanisms of drought response and their application in drought resistance improvement in plants. Cell. Mol. Life Sci. 72, 673–689. doi: 10.1007/s00018-014-1767-0, PMID: 25336153 PMC11113132

[B11] Folta K. M. Barbey C. R. (2019). The strawberry genome: A complicated past and promising future. Horticult. Res. 6, 34. doi: 10.1038/s41438-019-0101-7, PMID: 31645955 PMC6804783

[B12] Food and Agriculture Organization of the United Nations (FAO) (2023). FAOSTAT Agricultural Production Database. Available online at: https://www.fao.org/faostat/en/ (Accessed March, 2024).

[B13] Gao F. Li J. Li W. Shi S. Song S. Shen Y.. (2024). Abscisic acid and polyamines coordinately regulate strawberry drought responses. Plant Stress 11, 100387. doi: 10.1016/j.stress.2024.100387

[B14] Ghaderi N. Normohammadi S. Javadi T. (2015). Morpho-physiological responses of strawberry (Fragaria × ananassa) to exogenous salicylic acid application under drought stress. J. Agric. Sci. Technol. 17, 167–178.

[B15] Ghaderi N. Siosemardeh A. (2011). Response to drought stress of two strawberry cultivars (cv. Kurdistan and Selva). Horticult. Environment Biotechnol. 52, 6–12. doi: 10.1007/s13580-011-0019-6

[B16] Giampieri F. Forbes-Hernandez T. Y. Gasparrini M. Alvarez-Suarez J. M. Afrin S. Bompadre S.. (2015). Strawberry as a health promoter: An evidence-based review. Food Funct. 6, 1386–1398. doi: 10.1039/C5FO00147A, PMID: 25803191

[B17] Gonzalez-Fuentes J. A. Shackel K. Lieth J. H. Albornoz F. Benavides-Mendoza A. Evans R. Y. (2016). Diurnal root zone temperature variations affect strawberry water relations, growth, and fruit quality. Sci. Hortic. 203, 169–177. doi: 10.1016/j.scienta.2016.02.040

[B18] González-Villagra J. Ávila K. Gajardo H. A. Bravo L. A. Ribera-Fonseca A. Jorquera-Fontena E.. (2024). Diurnal high temperatures affect the physiological performance and fruit quality of highbush blueberry (Vaccinium corymbosum L.) cv. Legacy. Plants 13, 1846. doi: 10.3390/plants13131846, PMID: 38999686 PMC11244011

[B19] Kiliç T. (2023). Seed treatments with salicylic and succinic acid to mitigate drought stress in flowering kale cv. ‘Red Pigeon F1’. Sci. Hortic. 313, 111939. doi: 10.1016/j.scienta.2022.111939

[B20] Klamkowski K. Treder W. (2008). Response to drought stress of three strawberry cultivars grown under greenhouse conditions. J. Fruit Ornamental Plant Res. 16, 179–188.

[B21] Li Z. Zhang Y. Liu C. Gao Y. Han L. Chu H. (2022). Arbuscular mycorrhizal fungi contribute to reactive oxygen species homeostasis of Bombax ceiba L. under drought stress. Front. Microbiol. 13. doi: 10.3389/fmicb.2022.991781, PMID: 36204632 PMC9530913

[B22] Markwell J. Osterman J. C. Mitchell J. L. (1995). Calibration of the Minolta SPAD-502 leaf chlorophyll meter. Photosynthesis Res. 46, 467–472. doi: 10.1007/BF00032301, PMID: 24301641

[B23] Martínez-Ferri E. Soria C. Ariza M. T. Medina J. J. Miranda L. Domíguez P.. (2016). Water relations, growth and physiological response of seven strawberry cultivars (Fragaria × ananassa Duch.) to different water availability. Agric. Water Manage. 164, 73–82. doi: 10.1016/j.agwat.2015.08.008

[B24] Mishra N. Jiang C. Chen L. Paul A. Chatterjee A. Shen G. (2023). Achieving abiotic stress tolerance in plants through antioxidative defense mechanisms. Front. Plant Sci. 14. doi: 10.3389/fpls.2023.1110622, PMID: 37332720 PMC10272748

[B25] Muhammad I. Shalmani A. Ali M. Yang Q. H. Ahmad H. Li F. B. (2021). Mechanisms regulating the dynamics of photosynthesis under abiotic stresses. Front. Plant Sci. 11. doi: 10.3389/fpls.2020.615942, PMID: 33584756 PMC7876081

[B26] Nakabayashi R. Yonekura-Sakakibara K. Urano K. Suzuki M. Yamada Y. Nishizawa T.. (2014). Enhancement of oxidative and drought tolerance in Arabidopsis by overaccumulation of antioxidant flavonoids. Plant J. 77, 367–379. doi: 10.1111/tpj.12388, PMID: 24274116 PMC4282528

[B27] Nezhadahmadi A. Faruq G. Rashid K. (2015). The impact of drought stress on morphological and physiological parameters of three strawberry varieties in different growing conditions. Pakistan J. Agric. Sci. 52, 79–85.

[B28] Ödemiş B. Candemir D. K. Evrendilek F. (2020). Responses to drought stress levels of strawberry grown in greenhouse conditions. Hortic. Stud. 37, 113–122. doi: 10.16882/HortiS.738114

[B29] Pękal A. Pyrzynska K. (2013). Application of free radical diphenylpicrylhydrazyl (DPPH) to estimate the antioxidant capacity of food samples. Analytical Methods 5, 4288–4295. doi: 10.1039/c3ay40367j

[B30] Panda S. K. Gupta D. Patel M. Vyver C. V. D. Koyama H. (2024). Functionality of reactive oxygen species (ROS) in plants: Toxicity and control in Poaceae crops exposed to abiotic stress. Plants 13, 2071. doi: 10.3390/plants13152071, PMID: 39124190 PMC11313751

[B31] Peñuelas J. Savé R. Marfà O. Serrano L. (1992). Remotely measured canopy temperature of greenhouse strawberries as indicator of water status and yield under mild and very mild water stress conditions. Agric. For. Meteorol. 58, 63–77. doi: 10.1016/0168-1923(92)90104-W

[B32] Rahimi M. Kordrostami M. Mohamadhasani F. Safaei Chaeikar S. (2021). Antioxidant gene expression analysis and evaluation of total phenol content and oxygen-scavenging system in tea accessions under normal and drought stress conditions. BMC Plant Biol. 21, 447. doi: 10.1186/s12870-021-03195-2, PMID: 34706647 PMC8549219

[B33] Razavi F. Pollet B. Steppe K. Van Labeke M. C. (2008). Chlorophyll fluorescence as a tool for evaluation of drought stress in strawberry. Photosynthetica 46, 631–633. doi: 10.1007/s11099-008-0105-y

[B34] Şimşek Ouml;. (2024). Machine learning offers insights into the impact of *in vitro* drought stress on strawberry cultivars. Agriculture 14, 294. doi: 10.3390/agriculture14020294

[B35] Save R. Peñuelas J. Marfà O. Serrano L. (1993). Changes in leaf osmotic and elastic properties and canopy structure of strawberries under mild water stress. HortScience 28, 925–927. doi: 10.21273/HORTSCI.28.9.925

[B36] Sun C. Li X. Hu Y. Zhao P. Xu T. Sun J.. (2015). Proline, sugars, and antioxidant enzymes respond to drought stress in the leaves of strawberry plants. Horticultural Science and Technology 33(5), 625–632. doi: 10.7235/hort.2015.15054

[B37] Terry L. A. Chope G. A. Bordonaba J. G. (2008). Effect of water deficit irrigation on strawberry (*Fragaria* × *ananassa*) fruit quality In VI International Strawberry Symposium, 842 (pp. 839–842). Huelva, Spain: International Society for Horticultural Science (ISHS).

[B38] Tombesi S. Nardini A. Frioni T. Soccolini M. Zadra C. Farinelli D.. (2015). Stomatal closure is induced by hydraulic signals and maintained by ABA in drought-stressed grapevine. Sci. Rep. 5, 12449. doi: 10.1038/srep12449, PMID: 26207993 PMC4513549

[B39] Trenberth K. E. (2011). Changes in precipitation with climate change. Climate Res. 47, 123–138. doi: 10.3354/cr00953

[B40] Turner N. C. (1997). Further progress in crop water relations. Adv. Agron. 58, 293–338. doi: 10.1016/S0065-2113(08)60255-3

[B41] Ünal N. Okatan V. (2023). Effects of drought stress treatment on phytochemical contents of strawberry varieties. Sci. Hortic. 316, 112013. doi: 10.1016/j.scienta.2023.112013

[B42] Wilkinson S. Clephan A. L. Davies W. J. (2001). Rapid low temperature-induced stomatal closure occurs in cold-tolerant Commelina communis leaves but not in cold-sensitive tobacco leaves, via a mechanism that involves apoplastic calcium but not abscisic acid. Plant Physiol. 126, 1566–1578. doi: 10.1104/pp.126.4.1566, PMID: 11500555 PMC117156

[B43] Yang Y. J. Bi M. H. Nie Z. F. Jiang H. Liu X. D. Fang X. W.. (2021). Evolution of stomatal closure to optimize water-use efficiency in response to dehydration in ferns and seed plants. New Phytol. 230, 2001–2010. doi: 10.1111/nph.17253, PMID: 33586157

[B44] Yang X. Lu M. Wang Y. Wang Y. Liu Z. Chen S. (2021). Response mechanism of plants to drought stress. Horticulturae 7, 50. doi: 10.3390/horticulturae7030050

[B45] Zahedi S. M. Hosseini M. S. Hoveizeh N. F. Kadkhodaei S. Vaculík M. (2023). Physiological and biochemical responses of commercial strawberry cultivars under optimal and drought stress conditions. Plants 12, 496. doi: 10.3390/plants12030496, PMID: 36771578 PMC9919021

[B46] Zahedi S. M. Moharrami F. Sarikhani S. Padervand M. (2020). Selenium and silica nanostructure-based recovery of strawberry plants subjected to drought stress. Sci. Rep. 10, 17672. doi: 10.1038/s41598-020-74894-w, PMID: 33077742 PMC7572471

[B47] Zion Market Research (2023). Global Strawberry Market Analysis Report. Available online at: https://www.zionmarketresearch.com/ (Accessed March, 2024).

