# Correction: Screening of strawberry (*Fragaria × ananassa* Duch.) cultivars for drought tolerance based on physiological and biochemical responses under PEG-induced stress

**DOI:** 10.3389/fpls.2025.1712164

**Published:** 2025-10-14

**Authors:** Şule Hilal Attar, Duygu Ayvaz Sonmez, Azam Akbari, Doğan Ergün, Ömer Faruk Bilgin, Betül Yeşil, Merve Onur Bozkurt, Hayriye Yıldız Daşgan, Boran İkiz, Salih Kafkas, Bruno Mezzetti, Nesibe Ebru Kafkas

**Affiliations:** ^1^ Department of Horticulture, University of Çukurova, Faculty of Agriculture, Adana, Türkiye; ^2^ Yaltir Agricultural Products Inc., Adana, Türkiye; ^3^ Department of Horticulture, University of Siirt, Faculty of Agriculture, Siirt, Türkiye; ^4^ Department of Silviculture, Faculty of Forestry, Istanbul University-Cerrahpaşa, Bahçeköy, Sariyer, Istanbul, Türkiye; ^5^ Department of Agricultural, Food and Environmental Sciences, Universita Politecnica delle Marche, Ancona, Italy

**Keywords:** strawberry, drought stress, PEG-induced stress, physiological traits, biochemical markers, cultivar-specific response, antioxidant activity

The **Author contributions** statement was erroneously given as “ŞA: Data curation, Methodology, Investigation, Writing – original draft. DA: Investigation, Resources, Methodology, Writing – original draft. AA: Writing – original draft. DE: Data curation, Methodology, Writing – original draft, Investigation. ÖB: Writing – original draft, Investigation. BY: Writing – original draft, Investigation. MB: Writing – original draft, Resources. YD: Supervision, Writing – original draft, Investigation. Bİ: Investigation, Writing – original draft. SK: Resources, Writing – review & editing, Visualization. BM: Validation, Supervision, Conceptualization, Writing – original draft, Project administration. NK: Resources, Validation, Project administration, Writing – review & editing, Conceptualization, Supervision.”

The correct **Author contributions** statement is ŞA: Data curation, Methodology, Investigation, Writing – review & editing. DA: Investigation, Resources, Methodology, Writing – review & editing. AA: Data curation, Statistical data analysis, Visualization, Validation, Writing – original draft, Writing – review & editing. DE: Data curation, Methodology, Investigation, Writing – review & editing. ÖB: Investigation, Writing – review & editing. BY: Investigation, Writing – review & editing. MB: Resources, Writing – review & editing. YD: Supervision, Investigation, Writing – review & editing. Bİ: Investigation, Writing – review & editing. SK: Resources, Visualization, Writing – review & editing. BM: Validation, Supervision, Conceptualization, Project administration, Writing – review & editing. NK: Resources, Validation, Project administration, Conceptualization, Supervision, Writing – review & editing.

The original version of this article has been updated.

