# Supplementary material for: Screening of strawberry (Fragaria × ananassa Duch.) cultivars for drought tolerance based on physiological and biochemical responses under PEG-induced stress
Source: Front Plant Sci. 2025 Sep 15;16:1655320. doi: 10.3389/fpls.2025.1655320 (PMC12477223; doi:10.3389/fpls.2025.1655320)
Supplement: Supplementary file 1 [file Table1.docx]

**Table S1 Analysis of Variance (ANOVA) for Physiological, Biochemical, and Antioxidant Traits Under Different Treatments in Strawberry Cultivars**

| Trait | Year | Rep(Year) | Cultivar | Treatment | Cultivar × Treatment | Cultivar ×Year | Treatment ×Year | Cultivar × Treatment ×Year |
| --- | --- | --- | --- | --- | --- | --- | --- | --- |
| Df | 1 | 4 | 5 | 1 | 5 | 5 | 1 | 5 |
| Leaf Temp (°C) | 2390.86 ** | 1.03 * | 6.11 ** | 121.42 ** | 4.77 ** | 13.60 ** | 0.70 ns | 0.64 ns |
| Chlorophyll (µmol/m²) | 3336.08 ** | 4.82 ** | 68.41 ** | 144.78 ** | 18.70 ** | 36.63 ** | 1.15 ns | 1.60 ns |
| Leaf Number | 480.50 ** | 4.61 ns | 171.92 ** | 1200.50 ** | 6.53 ns | 159.07 ** | 56.89 ** | 14.86 * |
| RWC (%) | 4958.79 ** | 9.19 ns | 73.09 ** | 1968.09 ** | 26.22 ns | 83.45 ** | 281.11 ** | 26.22 ns |
| Photosynthetic Quantum | 0.1379 ** | 0.00028 ns | 0.0119 ** | 0.0696 ** | 0.0027 ** | 0.0195 ** | 0.00083 ns | 0.00031 ns |
| Dried Leaf Sucrose Content (%) | 39.04 ** | 0.0906 ns | 3.68 ** | 82.36 ** | 3.62 ** | 2.77 ** | 51.73 ** | 1.15 ** |
| Dried Leaf Glucose Content (%) | 70.35 ** | 0.0739 ns | 4.96 ** | 91.85 ** | 3.52 ** | 3.13 ** | 48.93 ** | 2.73 ** |
| Dried Leaf Xylose Content (%) | 0.0244 ** | 0.00278 ns | 0.0308 ** | 0.2833 ** | 0.0122 ** | 0.0089 ** | 0.0493 ** | 0.0199 ** |
| Dried Leaf Fructose Content (%) | 68.66 ** | 0.1839 * | 4.40 ** | 86.69 ** | 3.11 ** | 4.73 ** | 41.95 ** | 4.04 ** |
| Dried Leaf Total Sugar (%) | 532.62 ** | 0.2467 ns | 13.45 ** | 812.39 ** | 22.48 ** | 15.82 ** | 436.25 ** | 21.44 ** |
| Leaf Dry Matter Content (%) | 197.72 ** | 1.30 ns | 116.40 ** | 235.14 ** | 11.40 ns | 75.87 ** | 2.77 ns | 2.61 ns |
| Antioxidant Activity (%) | 962.72 ** | 0.157 ns | 128.55 ** | 43.77 ** | 2.34 ** | 142.55 ** | 9.28 ** | 0.85 ** |
| Free Radical Scavenging (%) | 52.15 ** | 0.137 ns | 114.59 ** | 81.45 ** | 1.70 ** | 158.76 ** | 4.36 ** | 2.02 ** |
| Succinic Acid Content (%) | 1091.54 ** | 0.1868 ns | 33.18 ** | 173.73 ** | 10.42 ** | 47.55 ** | 60.64 ** | 8.59 ** |

**Table S2 Analysis of Variance (ANOVA) for Physiological, Biochemical, and Antioxidant Traits Under Different Treatments in Strawberry Cultivars in the First Year**

| Source | Cultivar | | Treatment | | Cultivar × Treatment |
| --- | --- | --- | --- | --- | --- |
| DF | 14 | 1 | | 14 | |
| Leaf Temp (°C) | 2.87, *** | 46.57, *** | | 3.05, *** | |
| Chlorophyll (µmol/m)² | 19.71, *** | 33.14, *** | | 1.35, *** | |
| RWC (%) | 141.20, *** | 1437.83, *** | | 28.42, *** | |
| Photosynthetic Quantum Yield of Leaf | 0.00813, *** | 0.03503, *** | | 0.00094, *** | |
| Leaf number | 69.04, *** | 469.50, *** | | 5.26, *** | |
| Dried Leaf Sucrose Content (%) | 2.57, *** | 237.30, *** | | 2.17, *** | |
| Dried Leaf Glucose Content (%) | 4.38, *** | 361.96, *** | | 2.42, *** | |
| Dried Leaf Xylose Content (%) | 0.01842, *** | 0.29350, *** | | 0.01677*** | |
| Dried Leaf Fructose Content (%) | 4.39, *** | 259.47, *** | | 2.22, *** | |
| Dried Leaf Total Sugar (%) | 22.82, *** | 2357.83, *** | | 26.88, *** | |
| Ferric Reducing Antioxidant Power | 139.40, *** | 97.57, *** | | 2.91, *** | |
| Antioxidant Activity (%) | 141.48, *** | 138.19, *** | | 3.10, *** | |
| Free Radical Scavenging (%) | 6.38, *** | 81.08, *** | | 3.61, *** | |
| Leaf Dry Matter Content (%) | 19.41, *** | 430.65, *** | | 25.53, *** | |
| Dried Leaf Succinic Acid Content | 2.87, *** | 46.57, *** | | 3.05, *** | |

**Table S3 Analysis of Variance (ANOVA) for Physiological, Biochemical, and Antioxidant Traits Under Different Treatments in Strawberry Cultivars in the second year**

| Source | Cultivar | | Treatment | | Cultivar × Treatment |
| --- | --- | --- | --- | --- | --- |
| DF | 6 | 1 | | 6 | |
| Leaf Temp (°C) | 2.87, *** | 46.57, *** | | 3.05, *** | |
| Chlorophyll (µmol/m)² | 19.71, *** | 33.14, *** | | 1.35, *** | |
| RWC (%) | 141.20, *** | 1437.83, *** | | 28.42, *** | |
| Photosynthetic Quantum Yield of Leaf | 0.00813, *** | 0.03503, *** | | 0.00094, *** | |
| Leaf number | 69.04, *** | 469.50, *** | | 5.26, *** | |
| Dried Leaf Sucrose Content (%) | 2.57, *** | 237.30, *** | | 2.17, *** | |
| Dried Leaf Glucose Content (%) | 4.38, *** | 361.96, *** | | 2.42, *** | |
| Dried Leaf Xylose Content (%) | 0.01842, *** | 0.29350, *** | | 0.01677, *** | |
| Dried Leaf Fructose Content (%) | 4.39, *** | 259.47, *** | | 2.22, *** | |
| Dried Leaf Total Sugar (%) | 22.82, *** | 2357.83, *** | | 26.88, *** | |
| Antioxidant Activity (%) | 139.40, *** | 97.57, *** | | 2.91, *** | |
| Free Radical Scavenging (%) | 141.48, *** | 138.19, *** | | 3.10, *** | |
| Leaf Dry Matter Content (%) | 6.38, *** | 81.08, *** | | 3.61, *** | |
| Succinic Acid Content (%) | 19.41, *** | 430.65, *** | | 25.53, *** | |
